# Heparanase 2 (Hpa2) protects the exocrine pancreas from damages of Western diet and pancreatic cancer

**DOI:** 10.7150/ijbs.131032

**Published:** 2026-05-01

**Authors:** Yasmin Kayal, Malik Farhoud, Maali Odeh, Uri Barash, Inna Naroditsky, Rawan Bassal, Yasuhiko Nishioka, Ralph D Sanderson, Neta Ilan, Israel Vlodavsky

**Affiliations:** 1 Technion Integrated Cancer Center, Rappaport Faculty of Medicine, Technion, Haifa, Israel.; 2 Department of Pathology, Rambam Health Care Campus, Haifa, Israel.; 3 Department of Respiratory Medicine and Rheumatology, Tokushima University, Tokushima, Japan.; 4 Department of Pathology, University of Alabama at Birmingham, Birmingham, AL, USA.

**Keywords:** Heparanase 2, pancreas, high-fat diet, fatty pancreas, ADM, PanIN, MTORC

## Abstract

Fatty pancreas and pancreatitis are increasingly linked to Western dietary patterns and pancreatic cancer risk, yet intrinsic molecular mechanisms that preserve pancreatic identity under metabolic stress remain insufficiently defined. Heparanase-2 (Hpa2), a homolog of heparanase that lacks heparan sulfate-degrading activity, is clinically associated with favorable cancer outcomes, but its role in pancreatic homeostasis remains poorly defined. We fed wild-type (WT) and conditional Hpa2 knockout (Hpa2-KO) mice with a high-fat diet (HFD) and integrated histopathologic, transcriptomic, and proteomic analyses to define diet-dependent pancreatic responses. HFD elicited profound pancreatic remodeling selectively in Hpa2-KO mice, marked by massive fatty infiltration (fatty pancreas), extensive acinar-to-ductal metaplasia, β-islet hyperplasia, fibrosis, and pancreatic intraepithelial neoplasia (PanIN). These changes coincided with a decrease in acinar lineage identity, as reflected by marked suppression of GATA4, GATA6, MIST1, and PDX1. Systems-level analyses identified mTORC1 as a dominant signaling hub linking Hpa2 deficiency to metabolic reprogramming, with unanticipated effects on mitochondrial oxidative phosphorylation and ribosomal function. The results strongly imply that Hpa2 functions critically in preserving exocrine pancreatic identity and suppressing Western diet-driven pancreatic injury and neoplastic progression. Loss of Hpa2 advances diet-induced pancreatic disease, positioning Hpa2 as a key regulator of fatty pancreas, pancreatitis, and pancreatic cancer risk.

## Introduction

HPSE2, the gene encoding heparanase 2 (Hpa2), was cloned based on sequence homology to HPSE1 (heparanase) 1, an enzyme (endoglycosidase) that cleaves heparan sulfate (HS) side chains of heparan sulfate proteoglycans (HSPG) in the extracellular matrix (ECM) and cell surface. Cleavage of HS extracellularly by heparanase is thought to be unique, and results in structural alterations that make the ECM more prone to invasion by metastatic cells and cells of the immune system 2, 3. In addition, cleavage of HS by heparanase can release many growth factors, cytokines, chemokines, morphogens, and other mediators that are bound to HS as a storage depot, converting them into biologically active compounds 4, 5. Compelling evidence critically indicates that heparanase functions to promote tumorigenesis, thus raising the need for the development of heparanase inhibitors 6, 7, hoping to be implemented in clinical routine for cancer patients and possibly other human pathologies 3, 4, 8-10.

Little attention has been paid to Hpa2, and its roles in normal and pathological conditions remain largely obscure. Unlike heparanase, Hpa2 is thought to attenuate tumor progression, possibly due, in part, to inhibition of heparanase enzymatic activity 11-13. Unlike heparanase, Hpa2 is readily detected in normal epithelium, whereas its levels are decreased substantially in the resulting carcinomas 14-17, an expression pattern typical of tumor suppressors. Indeed, clinical studies revealed that high levels of Hpa2 are associated with prolonged survival of patients diagnosed with cervical, gastric, head and neck, liver, and pancreatic carcinomas vs patients bearing Hpa2-low tumors 11, 16-20, thus supporting the notion that Hap2 suppresses tumor progression. Experimentally, overexpression of Hpa2 in cancer cell lines resulted in smaller tumor xenografts, whereas silencing of Hpa2 resulted in bigger tumors 11, 16-18, 21-24. Altogether, these studies indicate that while Hpa2 and heparanase share a high degree of sequence homology and structural similarities 12, 25, they exert opposite functions in tumorigenesis 13, 26.

Decreased levels of Hpa2 were observed in several non-cancerous conditions. For example, a prominent decrease in the expression of Hpa2 was quantified in psoriasis lesions 27, and lower levels of Hpa2 were measured in the plasma of patients exhibiting severe symptoms of COVID-19 28 and in conditions of sepsis 29, 30. Another indication of Hpa2's involvement in inflammatory disorders emerged from our conditional Hpa2-KO mice, in which Hpa2 deficiency was associated with increased pancreatic inflammation 15. Moreover, the pancreas of Hpa2-KO male mice was found to be far more sensitive to cerulein 15, a well-established inducer of pancreatitis. This suggests that Hpa2 protects the pancreas against inflammation and pancreatitis.

Here, we fed wild-type (WT) and Hpa2-KO mice a high-fat diet (HFD), attempting to assess the significance of Hpa2 in liver physiology. While histological analysis clearly showed liver pathology, the severity of steatosis and liver fibrosis appeared comparable in WT-HFD and Hpa2-KO-HFD mice.

Remarkably, we found that HFD elicited severe alterations in the morphology of the pancreas of male Hpa2-KO mice, resulting in massive accumulation of fat cells (fatty pancreas) and cells undergoing acinar-to-ductal metaplasia (ADM), tissue fibrosis, and hyperplasia of beta islets. This strongly implies that Hpa2 plays a critical role in protecting the exocrine pancreas from damage caused by a high-fat, high-sugar (Western) diet.

## Materials and Methods

**Conditional Hpa2-KO mice.** HPSE2^fl^ mice (on C57BL/6 background) were described previously 15. Briefly, for the generation of the HPSE2 conditional knockout allele (HPSE2^fl^), the Neo cassette was flanked by self-deletion anchor (SDA) sites, and the cKO region was flanked by loxP sites, directed to the introns flanking exon 5. B6.Cg-Tg (CAG-cre/Esr1*) 5Amc/J mice were purchased from Jackson (JAX Stock No:004682). These CAGGCre-ERTM transgenic mice have a tamoxifen-inducible Cre-mediated recombination system driven by the chicken beta-actin promoter/enhancer coupled with the cytomegalovirus (CMV) immediate-early enhancer. When bred with mice carrying loxP-flanked sequences, tamoxifen-inducible Cre-mediated recombination results in deletion of the floxed sequences in a wide range of cells/tissues in the offspring. Cre activation was performed via administration (i.p) of tamoxifen (0.1 ml; Sigma T5648, 20 mg/ml dissolved in corn oil) every day for 4 days, resulting in the removal of exon five and the disruption of HPSE2 open reading frame. Tamoxifen was administered to 5-6-week-old Cre^+^ HPSE2^fl^ mice and to age-matched control C57BL/6 mice. PCR validated HPSE2 gene editing by Cre activation. For experiments, we used 3-month-old mice (typically 6 weeks after tamoxifen administration).

**Housing and diets**. *wt* C57BL/6 and Hpa2-KO mice (typically 10-12 weeks old; 22-25 g) were housed in a specific pathogen-free environment, maintained in a temperature-controlled environment with a 12-hour light/12-hour dark cycle, and provided with ad libitum access to water and food. The mice were fed two different diets. The low-fat, low-sucrose diet was standard cereal-based (soy, wheat, corn) mouse chow (Altromin,132**4**). Its composition (wt/wt) was **11**% fat, **65**% carbohydrate, and **24**% protein (3**2**2 kcal per 100 g). The HF-HS diet was a milk fat-based diet (TD.88137; Harlan Laboratories Inc., Indianapolis, IN, USA). Its composition was (wt/wt): 21.2% fat (60% saturated fatty acids), 49.1% carbohydrate (34.1% sucrose plus 15% corn starch), and 17.3% protein. Fats and carbohydrates provided 42% and 42.7 % of the calories, respectively (Supplementary Table 1), and the diet yielded 450 kcal per 100 g. Body weight was monitored once a week.

**Histopathological analyses.** Mouse pancreas and liver tissues collected from at least 5 mice were fixed with 4% paraformaldehyde and embedded in paraffin using standard protocols. Paraffin sections (5 μm) were stained with hematoxylin and eosin (H&E), Masson's trichrome, Sirius red, and alcian blue, essentially as described 15, 31. Specimens were evaluated and blindly scored by an expert pathologist. A total of at least five high-power fields in each pancreatic section, prepared from the pancreata of 5-7 mice, were evaluated for acinar cell hypertrophy, edema, acinar-to-ductal metaplasia (ADM), acinar-to-adipocytes trans-differentiation (AAT), beta islets hyperplasia, tissue fibrosis, and pancreatic intraepithelial neoplasia (PanIN). Paraffin-embedded 5 µm sections were immunostained with the indicated antibody using the Envision kit, according to the manufacturer's (Dako; Santa Clara, CA, USA) instructions, as previously described 15, 18, 21, 23. Staining intensity was quantified by Image Pro software, essentially as described 15.

**Antibodies and reagents**. Anti-heparanase antibody (733) was described previously 32. Anti-alpha-amylase (sc-166349), anti-PPAR ɣ (sc-7273), anti-Cox2 (sc-19999), anti-ERK2 (sc-154) and anti-cytokeratin 19 (sc-376126) antibodies were purchased from Santa Cruz Biotechnology; anti-Sox9 (#82630), anti-perilipin-1 (#9349), anti-phopsho-Acc (#11818) and anti-Mist1 (#14896) antibodies were purchased from Cell Signaling; anti-Ki67 (ab833), Anti-GRP78 (Bip) (ab109659), anti-GATA4 (ab307823) and anti-insulin antibodies were purchased from Abcam; anti CD45R (a B cell marker) was purchased from BioLegend (cat:103206) and anti F4/80 (MCA497GA; a marker of mouse macrophages) antibody was from Bio-Rad. Anti-actin (clone AC-74) and anti-smooth muscle actin (SMA; clone 1A4) antibodies, tamoxifen, alcian blue 8GX, and safranin were purchased from Sigma. Anti-cleaved LC3A antibody was purchased from Abgent (AP1805a).

**Real time-PCR**. Real-time-PCR analyses were performed using ABI PRISM 7000 Sequence Detection System employing SYBR Green PCR Master Mix (Applied Biosystems, Foster City, CA), essentially as described 15, 18.

**Proteomic analysis**. Proteomic analysis was performed on proteins extracted from 10-micron paraffin sections. Briefly, pancreas sections were deparaffinized using n-Hexan for 30 min (RT), followed by methanol addition and phase separation. Following centrifugation, the upper phase was discarded, and the lower phase was dried, washed once with 50% ethanol, and dried again. Protein pellets were then dissolved in 8.5 M urea, 400 mM ammonium bicarbonate, and 10 mM DTT. Protein amount was estimated using Bradford readings. The samples were reduced (60 ºC for 30 min), modified with 35.2 mM iodoacetamide in 100 mM ammonium bicarbonate (room temp for 30 min in the dark), and digested in 1.5 M urea, 66 mM ammonium bicarbonate with modified trypsin (Promega), overnight at 37 °C in a 1:50 (M/M) enzyme-to-substrate ratio. A second trypsinization was performed for 4 hours at a 1:100 (M/M) enzyme-to-substrate ratio. The tryptic peptides were desalted using Oasis HLB 96-well µElution Plate (Waters), dried, and re-suspended in 0.1% Formic acid in 2% acetonitrile**.** The resulting peptides were analyzed by LC-MS/MS using an Exploris 480 mass spectrometer (Thermo) fitted with a capillary HPLC (Vanquish, Thermo Scientific). The peptides were loaded in solvent A (0.1% formic acid in water) on a C18 reversed phase analytical column (Ionoptics, AUR3-25075C18-XT, 25cm x 75um ID, 1.7um). The peptide mixture was resolved using a 6-34% linear gradient of solvent B (80% acetonitrile with 0.1% formic acid in water) for over 2 hours. Mass spectrometry was performed in a positive mode using repetitively full MS scan (m/z 380-985, resolution 120,000) followed by data-independent acquisition (DIA) scans (10 Da isolation windows with 1 m/z overlap, and resolution 30,000). The mass spectrometry data were analyzed using the DIA-NN software version 2.0 (1, 2) searching against the Human and the Plasmodium falciparum proteome from the Uniprot database, with minimal peptide length set to 7, maximum number of missed cleavages set to 1, cysteine carbamidomethylation enabled as a fixed modification, and protein N-terminal acetylation enabled as a variable modification. Peptide- and protein-level false discovery rates (FDRs) were filtered to 1% and subjected to proteomic analysis. Briefly, proteins were trypsinized and analyzed by tandem mass spectrometry data-independent acquisition (MSMS DIA) utilizing Orbitrap Exploris 480 Mass Spectrometer (Thermo). The data was analyzed and quantified by the DIA-NN automated software suite for DIA of proteomics data 33, 34.

**RNAseq analysis**. RNAseq analysis was carried out by the Genomics Core Facility of the Rappaport Faculty of Medicine, Technion, essentially as described previously 35. Briefly, RNA-seq libraries were constructed simultaneously using the NEBNext Ultra II Directional RNA Library Prep Kit for Illumina, following the manufacturer's protocol (NEB, cat. no. E7760). For bioinformatics analysis, single reads (100 bps) were aligned to the mouse (mm10) reference (mm10-2020-A) using STAR (V2.5.3a). The number of reads per gene was evaluated using HTSeq-count (v**2**.**0**.2). Normalization and differential expression analyses were conducted using DESeq2 R package (v1.3**6**.0). The similarity between samples was evaluated within DESeq2 package using the Euclidean distance matrix and a principal component analysis (PCA). Gene set enrichment analysis (GSEA) was performed using GSEA graphical user interface for Windows (v4.3.2). p-adj = 0.01 was applied in the analyses of DEG.

**Statistics.** Data are presented as means ± SEM. Statistical significance was analyzed by a 2-tailed Student's *t*-test. Values of p ≤ 0.05 were considered significant and designated as follow: *, p ≤0.05; **, p ≤ 0.01; ***, p ≤ 0.001; ****, p ≤ 0.0001. Data sets passed D'Agostino-Pearson normality (GraphPad Prism 5 utility software).

## Results

**Hpa2-deficient pancreas is highly sensitive to HFD**. We have reported recently that high levels of Hpa2 are associated with prolonged survival time of patients diagnosed with hepatocellular carcinoma 19. To further examine the role of Hpa2 in liver biology, we utilized our conditional Hpa2-KO mouse model 15. Control (wt) and Hpa2-KO mice were fed with regular mouse chow (11% fat, 24% protein) or high fat (Western) diet (HFD) chow formulation, enriched with high fat (42% of calories) and carbohydrates (42.7% of calories; 34.1% sucrose plus 15% corn starch) and only 17.3% protein (TEKLAD 88137). Mice were fed an HFD for 13 weeks, a time point known to elicit mild pathologies (i.e., moderate-to-advanced hepatic steatosis, along with early signs of liver injury and inflammation). Male mice were used for the experiments. Body weight, liver-to-body weight ratio, blood ALT, and blood cholesterol parameters were significantly increased in mice fed with HFD vs control or standard chow, but comparable values were measured in *wt* and Hpa2-KO mice (Supplementary Fig. 1A-E). Histological analysis clearly presented liver pathology, but the severity of steatosis and liver fibrosis (Sirius red staining) appeared similar in *wt*-HFD and KO-HFD mice (Supplementary Fig. 1F).

**Figure 1:**
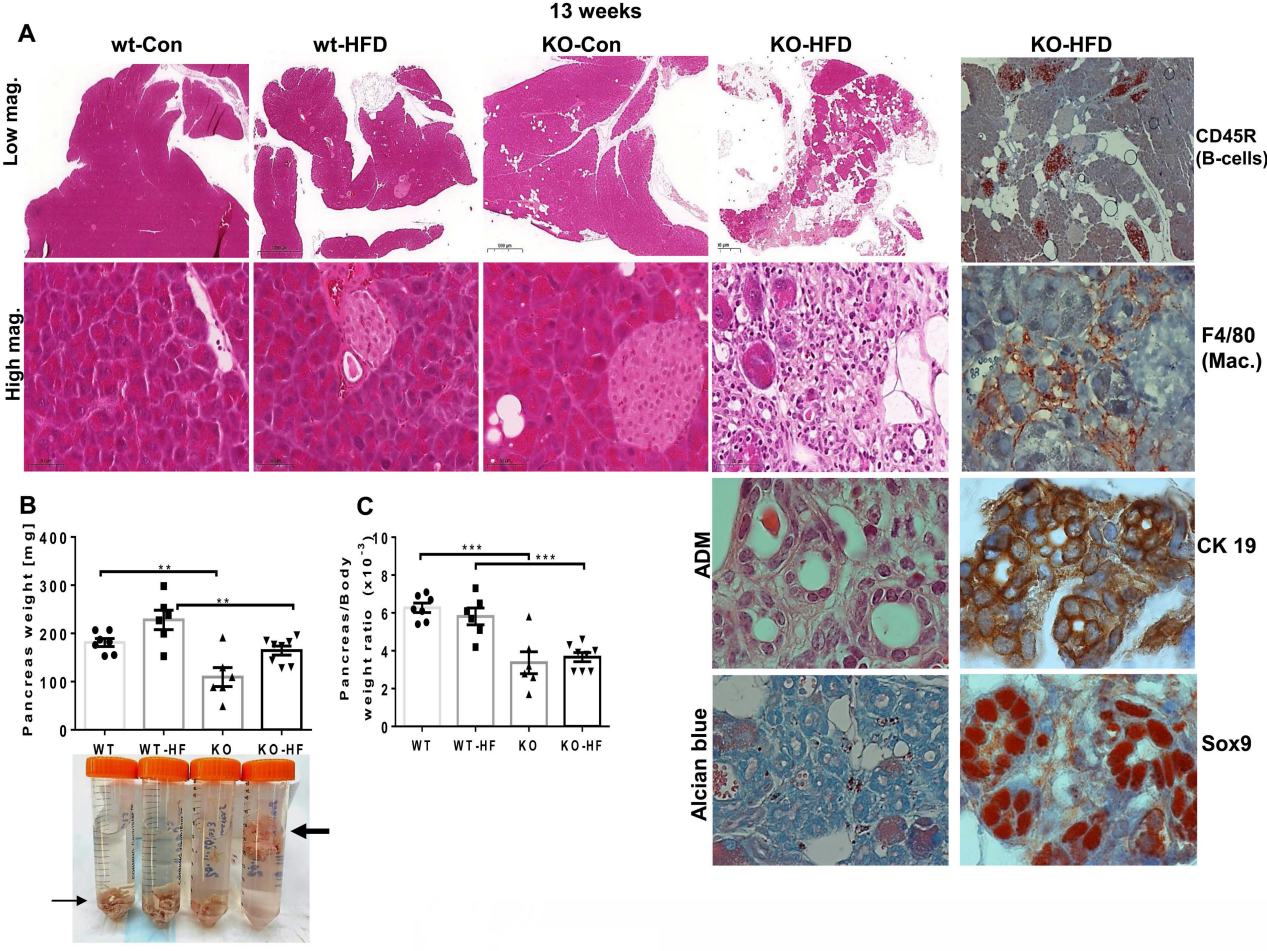
Hpa2-KO pancreas responds vigorously to HFD. WT and Hpa2-KO mice were fed regular mouse chow as a control (Con) or HFD for 13 weeks. Mice were then sacrificed, and pancreatic tissues were collected, weighed, and fixed in formalin for histological and immunohistological examinations. Shown are representative images of hematoxylin & eosin (HE) at low (x2.5; **A**, upper panels) and high (x50; **A**, second panels) magnifications. Acinar to ductal metaplasia (ADM) is shown at high (x200) magnification (second right, third panel). Slides were also stained for alcian blue (pH 5.8) that stains HS (second right, lower panel) or were subjected to immunostaining applying antibodies directed against CD45R (B cells), F4/80 (macrophages), cytokeratin 19 (CK 19), and Sox9 (right panels, respectively), the latter are measures of ADM. Pancreas weight and pancreas to body weight values are shown in **B**, **C**, respectively. Note that Hpa2-KO-HFD pancreas floats on the formalin fixative (**B**, lower panel, thick arrow).

**Figure 2:**
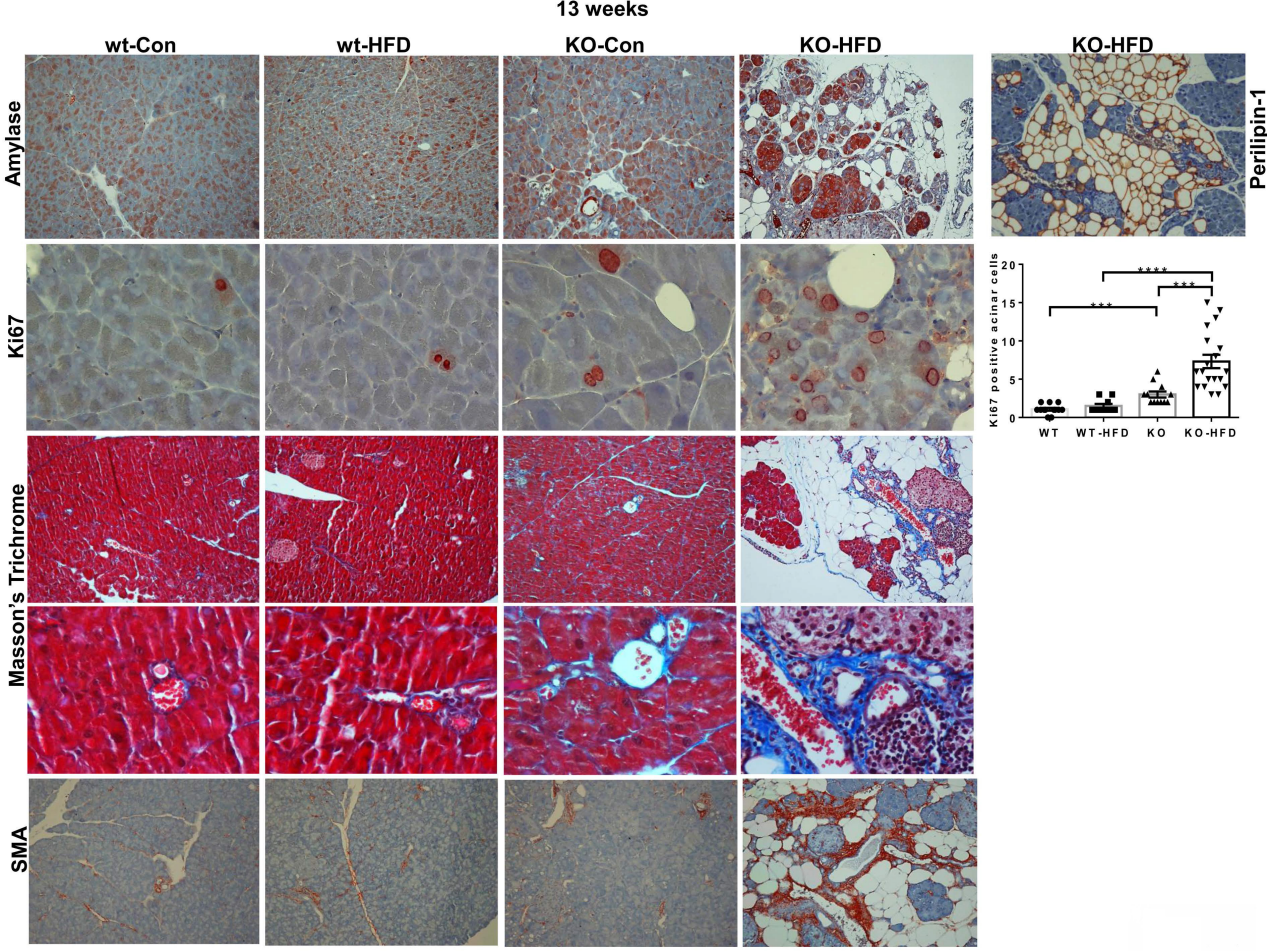
Hpa2-KO pancreas is fatty and fibrotic. WT and Hpa2-KO mice were fed regular mouse chow as control (Con) or HFD for 13 weeks. Mice were then sacrificed, and pancreatic tissues were collected, fixed in formalin, and subjected to histological and immunohistological examinations. Shown are representative images of immunostaining for alpha amylase (upper panels; original magnification x10) and Ki67 (second panels; original magnification x100). Quantification of Ki67-positive cells is shown graphically on the right panel. Immunostaining of Hpa2-KO pancreas section for perilipin-1 that labels adipocytes is shown in the upper right panel (original magnification x10). 5-micron sections were also stained with Masson's Trichrome reagent that labels collagens in blue (third and fourth panels; original magnification x10 and x100, respectively). 5-micron sections were similarly subjected to immunostaining with an anti-SMA antibody (lower panels; original magnification x100).

Given our recent publication, revealing a critical role for Hpa2 in the adult pancreas 15, we subsequently examined this tissue in mice fed with HFD. Surprisingly, we found that HFD elicited severe alterations in the morphology of the pancreas of male Hpa2-KO mice (Fig. 1). The pancreas of Hpa2-KO control male mice seems relatively preserved (Fig. 1A, KO-Con) yet appeared smaller by weight (atrophy) (Fig. 1B, C; p = 0.0005), in agreement with previous results 15. In striking contrast, Hpa2-KO mice fed with HFD exhibited severe morphological and cellular alterations (Fig. 1A, KO-HFD). These included **i.** massive accumulation of fat cells and cells undergoing acinar-to-ductal metaplasia (ADM) that are stained strongly for cytokeratin (CK)19, Sry-related high-mobility group box 9 (Sox9), and HS (Alcian blue), on the expense of the exocrine pancreas (acinar cells; Fig. 1A, KO-HFD), and **ii.** chronic inflammation evident by foci of B-cells (Fig. 1A, rightmost panel; CD45R), and accumulation of macrophages (Fig. 1A, rightmost panel; F4/80). Loss of acinar cells in Hpa2-KO HFD pancreas is apparent by decreased number of amylase-positive cells (Fig. 2, upper panels) and extensive staining of perilipin-1 that marks fat cells (Fig. 2, upper right). The fatty nature of the KO-HFD pancreas emerged from the gross observation that these pancreatic tissues float when placed in formalin for fixation (Fig. 1B, lower panel, arrow). Homeostasis of KO-HFD pancreas is apparently maintained by increased alpha-amylase expression by the remaining acinar cells (Fig. 2, KO-HFD, upper panel), and increased proliferation (i.e., Ki67 staining) of these cells (Fig. 2, second right panel, KO-HFD; 3±0.4 vs 7.3±0.8 for KO-Con vs KO-HFD; p = 0.0008). In addition, the KO-HFD pancreas appeared fibrotic, evident by increased collagen deposition underneath structures of ADM and blood vessels (KO-HFD; Fig. 2, Masson's Trichrome, blue) and a marked increase in the staining for smooth muscle actin (SMA; Fig. 2, lower panel). Thus, the Hpa2-KO-HFD pancreas exhibits a unique morphology that combines fatty, inflamed, and fibrotic tissue, the latter characterizes pancreatic tumors. Together, HFD converts the relatively preserved morphology of Hpa2-KO male pancreas into the morphology described previously for female Hpa2-KO pancreas, or the pancreas of male mice treated with cerulein 15.

**Figure 3:**
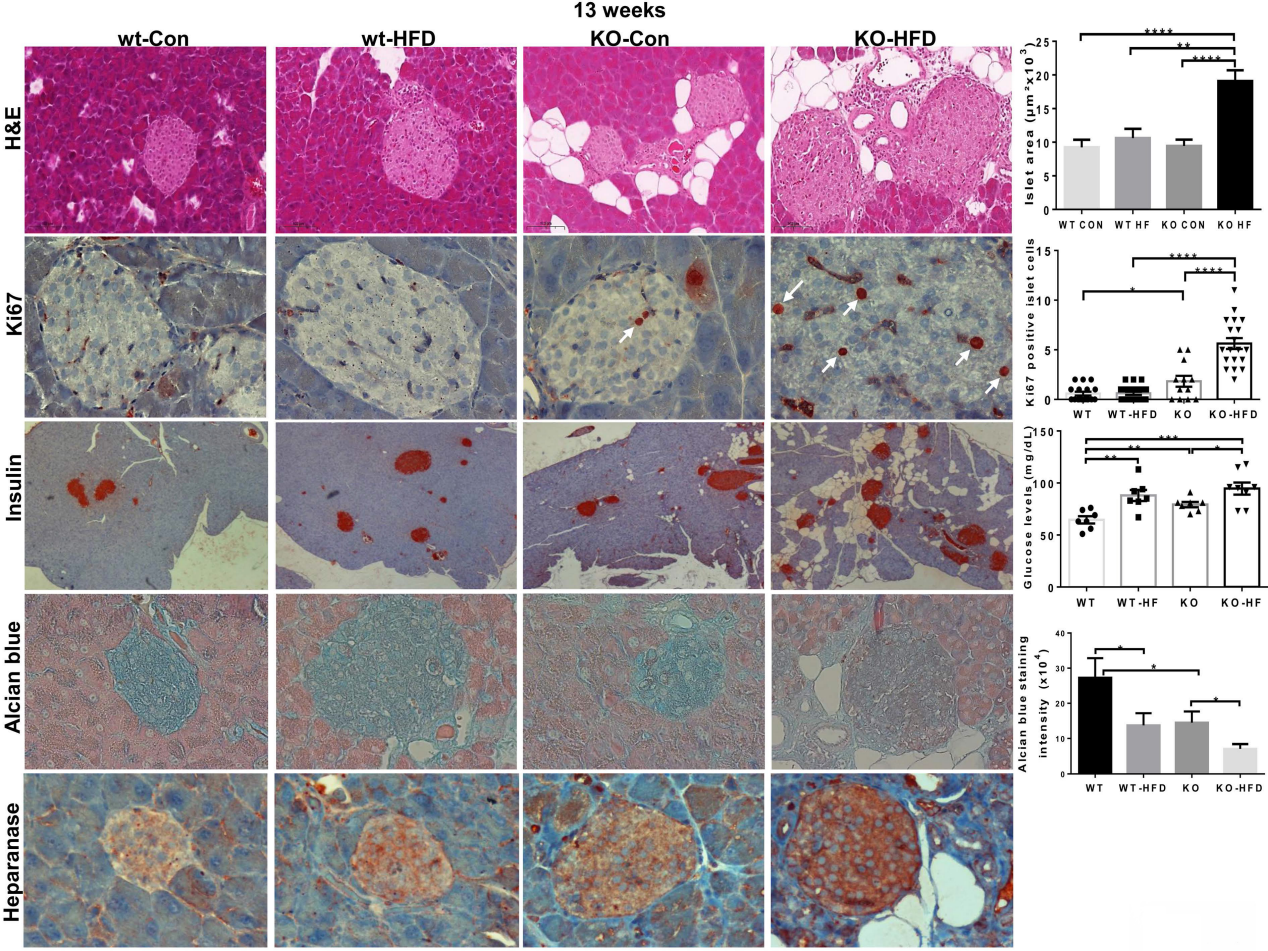
Hyperplasia of beta islets in Hpa2-KO pancreas following HFD. WT and Hpa2-KO mice were fed regular mouse chow (Con) or HFD for 13 weeks. Mice were then sacrificed, and pancreatic tissues were collected, fixed in formalin, and subjected to histological and immunohistological examinations. Shown are representative images of hematoxylin & eosin (HE) of beta islets (upper panels; original magnification x100). Quantification of beta islets area is shown graphically in the upper right panel. 5-micron sections were stained for Ki67 (white arrows; second panels; original magnification x100). Quantification of Ki67-positive cells in beta islets is shown graphically in the second right panel. Immunostaining for insulin is shown in the third panels (original magnification x10). Levels of blood glucose are shown graphically in the third right-most panel. 5-micron sections were also stained with alcian blue, which labels HS in blue (fourth panels; original magnification x100). Quantification of color intensity in beta islets is shown graphically in the rightmost, fourth panel. 5-micron sections were similarly subjected to immunostaining applying anti-heparanase antibody (lower panels; original magnification x100).

In addition, we observed previously unrecognized features, namely hyperplasia of beta islets in Hpa2-KO mice fed with HFD. This was concluded by histological analyses and measurements of the area of beta islets (Fig. 3, KO-HFD, upper panels;10.6±1.3x10^3^ vs 19.1±1.6x10^3^ µM^2^ for wt-HFD and KO-HFD, respectively; p = 0.0007), increased proliferation of beta cells (i.e., Ki67 staining; Fig. 3, KO-HFD, second panels; 0.6±0.1 vs 5.6±0.5 Ki67-positive cells/high power field in wt-HFD and KO-HFD pancreas, respectively; p ≤ 0.0001), and by insulin staining (Fig. 3, KO-HFD, third panels). Notably, blood glucose levels after fasting (18 hours) appeared comparable in all 4 groups (Fig. 3, third right). Of note, HS content in beta islets appeared to be decreased (Fig. 3, fourth panels), likely due to increased heparanase levels and activity in the absence of its endogenous inhibitor, Hpa2 (Fig. 3, lower panels). These results strongly imply that Hpa2 plays a critical role in protecting the exocrine pancreas from overload of fatty acids, mostly saturated fatty acids (> 60% of total fatty acids in this formulation) and/or carbohydrates (i.e., high content of sucrose).

**Hpa2-KO pancreas turns fatty already after 3 days of HFD**. To better define the timing of the robust and unexpected effect of HFD, we exposed Hpa2-KO male mice to HFD for decreasing durations. Interestingly, we found comparable effects of HFD on male Hpa2-KO pancreas following 8 weeks (Supplementary Fig. 2), 4 weeks (Supplementary Fig. 3), and 1 week (Supplementary Fig. 4) of this diet.

**Figure 4:**
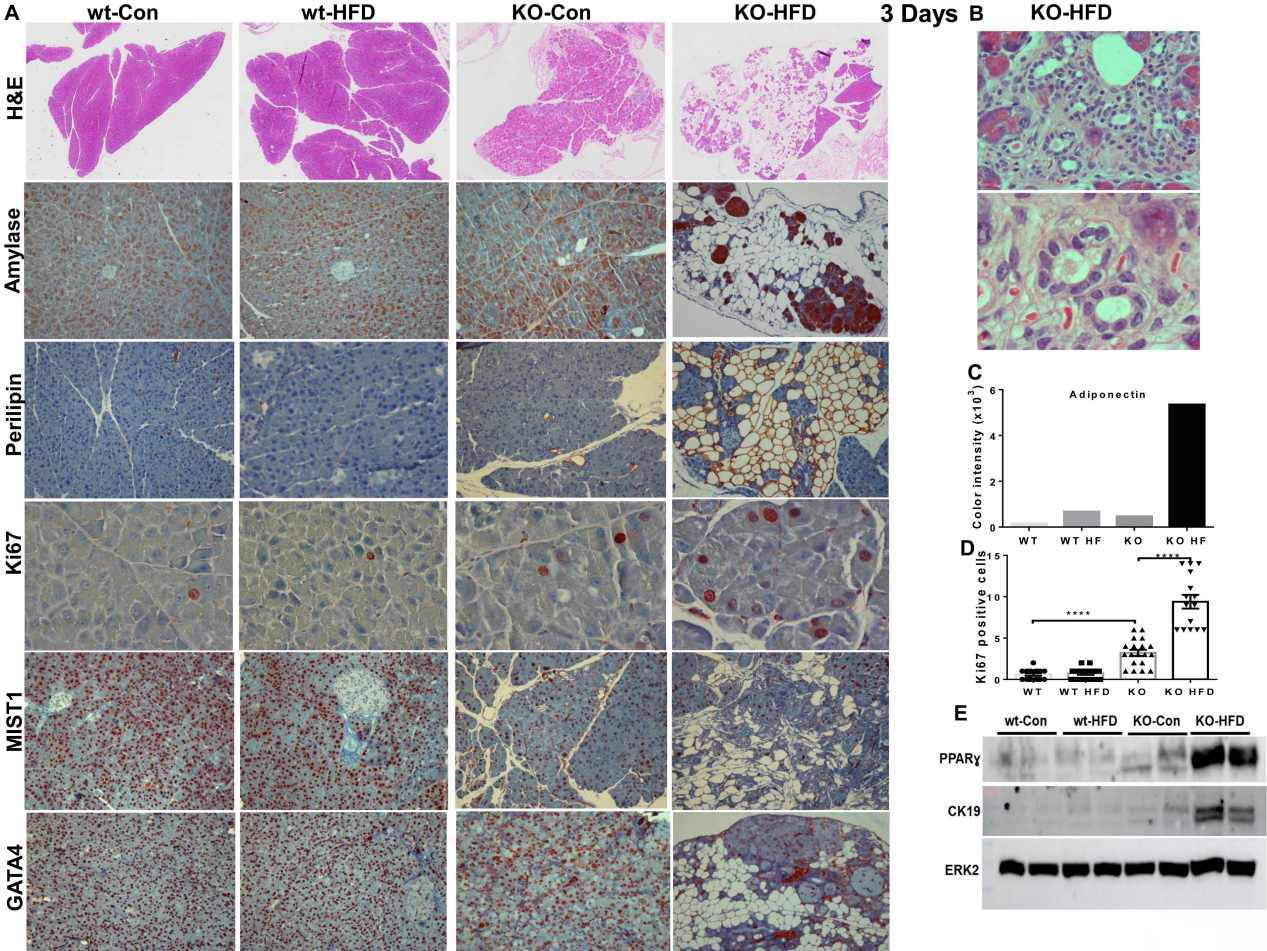
Three days of HFD are sufficient to elicit fatty pancreas in Hpa2-KO mice. **A, B**. Histology and immunostaining. WT and Hpa2-KO mice were fed regular mouse chow as control (Con) or HFD for 3 days. Mice were then sacrificed, and pancreatic tissues were collected, fixed in formalin, and subjected to histological and immunohistological examinations. Shown are representative images of hematoxylin & eosin (HE, upper panels; original magnifications x2.5). Acinar to ductal metaplasia in the pancreas of Hpa2-KO mice fed HFD is shown at higher magnifications in **B** (original magnifications x100, x250). 5-microm sections were subjected to immunostaining applying anti-alpha-amylase (second panels; original magnifications x25) and anti-perilipin-1 (third panels; original magnifications x25). Expression of adiponectin as a measure of adipocytes is shown graphically in **C**. Sections were also stained with anti-Ki67 (fourth panels; original magnifications x100) antibody. Quantification of Ki67-Positive cells is shown in **D**. Immunostaining for MIST1 and GATA4 (transcription factors that are important to the development and differentiation of the exocrine pancreas) is shown in the fifth and sixth panels, respectively (original magnifications x10). Note that accumulation of fat cells, ADM, and increased acinar cell proliferation are associated with decreased MIST1 and GATA4 expression as early as 3 days on HFD. **E**. Immunoblotting. Extracts of the indicated pancreas (each lane represents a pool of pancreas tissue extracted from 2-3 mice) were subjected to immunoblotting, applying antibodies directed against PPARɣ (upper panel), cytokeratin 19 (CK19, second panel), and ERK2 (lower panel as loading control).

**Figure 5:**
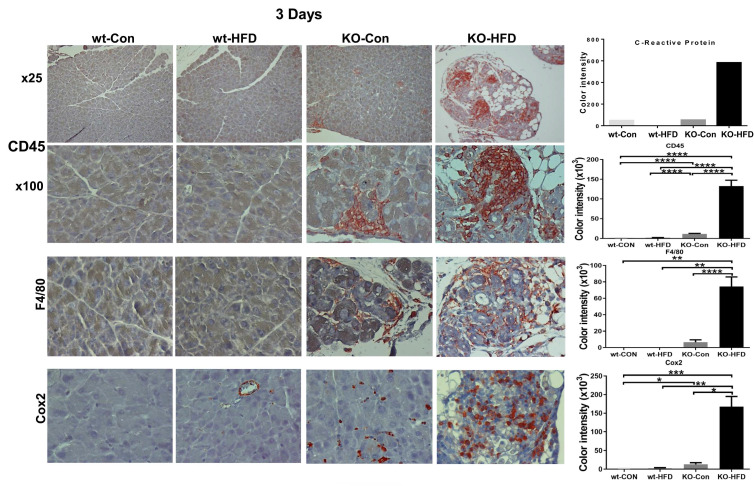
HFD promotes inflammation of Hpa2-KO pancreas within 3 days. WT and Hpa2-KO mice were fed regular mouse chow as control (Con) or HFD for 3 days. Mice were then sacrificed, and pancreatic tissues were collected, fixed, and subjected to immunostaining. Shown are representative images of immunostaining for CD45 at low (upper panels; original magnification x10) and high (second panels; x 100) magnifications, F4/80 (i.e., macrophages; third panels; original magnification x100), and Cox2 (lower panels; original magnification x100). Quantification of C-reactive protein (CRP) as a measure of inflammation, and quantification of the staining for CD45, F4/80, and Cox2 are shown graphically in the right panels. Note a substantial increase in the inflammation of Hpa2-KO pancreas following HFD for only three days.

**Figure 6:**
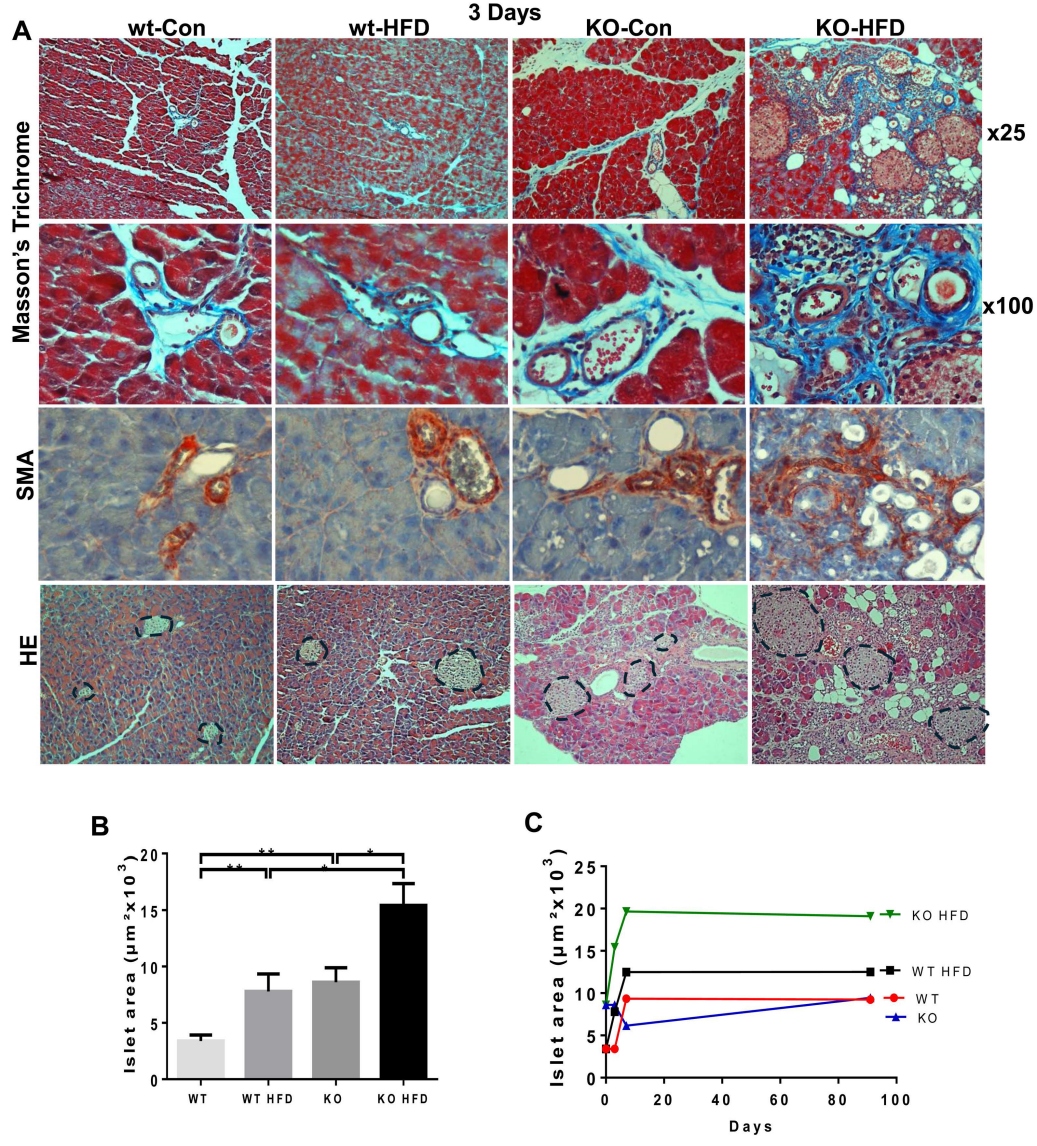
HFD promotes fibrosis of Hpa2-KO pancreas within 3 days. WT and Hpa2-KO mice were fed regular mouse chow as control (Con) or HFD for 3 days. Mice were then sacrificed, and pancreatic tissues were collected, fixed, and subjected to histological examinations. Shown are representative images of staining for Masson's Trichrome at low (x10; **A**, upper panels) and high (x100; **A**, second panels) magnifications. Immunostaining for αSMA is shown in **A**, third panels. Note that in wt-Con, wt-HFD, and KO-Con, collagen and SMA-positive staining is restricted primarily to blood vessels. In contrast, in KO-HFD, staining is widespread across ADM structures, resulting in a fibrotic pancreas. HE staining is shown in the lower panels of A; Beta-islets are circled. The average area of beta islets in each group of mice is shown graphically in **B**. Comparison between beta islets areas after 3 days, 1 week, and 13 weeks of HFD is shown graphically in **C**. Note the increase in beta islets area in Hpa2-KO mice fed with HFD.

In fact, we found that 3 days of HFD results in morphological alterations comparable to those observed after 13 weeks of HFD, as evidenced by HE staining (Fig. 4A, upper panels; Fig. 4B) and immunostaining for alpha-amylase (Fig. 4A, second panels). We further used perilipin-1 (Fig. 4A, third panel), adiponectin (Fig. 4C), and PPARɣ (Fig. 4E, upper panel; Supplementary Fig. 5A) as measures for fat cells and observed nearly 10-fold increase in adipocyte content in KO-HFD vs KO-control pancreas. This was accompanied by the decreased number of amylase-positive acinar cells (Fig. 4, second panels), balanced by a significant increase in acinar cell proliferation (Ki67; Fig. 4A, fourth panels; Fig. 4D; 0.7±0.1 vs 9.4±0.8 for KO-Con vs KO-HFD; p=0.0001). Notably, these alterations correlate with a noticeable decrease in staining intensities of basic helix-loop-helix family member A15 (MIST1; Fig. 4A, fifth panels) and GATA4 (Fig. 4A, lower panels), acinar-specific transcription factors that function to sustain acinar differentiation and to protect the pancreas from cell transformation 36-38. In addition, we observed a markedly increased number of CD45-positive cells (i.e., immune cells) and F4/80-positive cells (macrophages) in KO-HFD vs KO-control pancreas (Fig. 5, upper panels) in mice fed 3 days with HFD. We also found a pronounced increase in the number of Cox2-positive cells (Fig. 5, lower panel) and used C-reactive protein (CRP) as a measure of inflammation levels in the Hpa2-KO pancreas after 3 days of HFD feeding 39. We observed over a 10-fold increase in CRP levels in KO-HFD vs KO-Con (Fig. 5, upper right), and similar increases were quantified for CD45, F4/80, and cox2 in the pancreas of the Hpa2-KO-HFD mice (Fig. 5, right panels), altogether indicating a prominent increase in pancreatic inflammation in Hpa2-KO mice fed with HFD vs KO-Con. Furthermore, 3 days of HFD resulted in a marked increase in fibrosis of the Hpa2-KO pancreas, evident by Masson's Trichrome (Fig. 6, upper panels) and SMA staining (Fig. 6, third panels), to a magnitude comparable to that of fibrosis observed by 13 weeks of HFD (Fig. 2). This is a unique and quite remarkable output of HFD, clearly displaying the susceptibility of the Hpa2-KO pancreas to metabolic (HFD) overload. In addition, we found that HFD for 3 days already elicits enlargement of beta islets (Fig. 6, lower panel; Fig. 6B). Comparing beta islets areas at early (3 days and 1 week) vs late (13 weeks) time points revealed that beta islets area reaches its maximum at 1 week, and this enlargement is maintained throughout the course of 13 weeks (Fig. 6, C). We applied immunoblotting for cytokeratin (CK)19 as a measure for ADM (Fig. 4E, second panel) and found over 9-fold increase of CK19 levels in KO-HFD vs KO-Con pancreas (Supplementary Fig. 5B). Altogether, the results show that HFD-fed Hpa2-KO mice develop, within 3 days, fatty pancreas that is also fibrotic and undergoes extensive ADM and inflammation.

**Proteomic and transcriptomic analyses: involvement of the MTORC1 signaling pathway**. To explore the effects of Hpa2 deficiency and HFD on the pancreas at the molecular level, we first utilized proteomic analysis. For this purpose, we extracted proteins from pancreatic 10-micron paraffin sections of WT and Hpa2-KO mice fed normal (Control) or HFD for 3 days. The analysis shows decreased levels of acinar enzymes in KO-HFD vs KO-Con pancreas (i.e., carboxypeptidase A1, A2, B; trypsin, phospholipase A2; chymotrypsinogen B, Supplementary Table 2), and a substantial decrease in the levels of pancreatic transcription factors, including GATA4, GATA6, and duodenal homeobox 1 (PDX1) (Supplementary Table 2) in KO-HFD vs KO-Con pancreas, in agreement with the immunostaining results (Fig. 4). Moreover, as expected, the analysis revealed increased levels of adipocyte markers such as perilipin 4, long-chain fatty acid transport protein, phospholipid transfer protein, and others (Supplementary Table. 2), thus confirming and further expanding the fatty nature of KO-HFD pancreas. KEGG pathway analysis further revealed increased ECM-receptor interactions and focal adhesion in KO-HFD vs WT-HFD pancreas (Supplementary Fig. 6A), possibly reflecting the strong fibrotic phenotype of KO-HFD pancreas (Figs. 2, 6). Notably, the most significant alteration according to the KEGG analysis was a decrease in ribosome in KO-HFD vs WT-HFD (Supplementary Fig. 6B) and decreased levels of multiple cytosolic and mitochondrial ribosomal protein subunits in KO-HFD vs KO-Con (Supplementary Table 2) pancreas.

**Figure 7:**
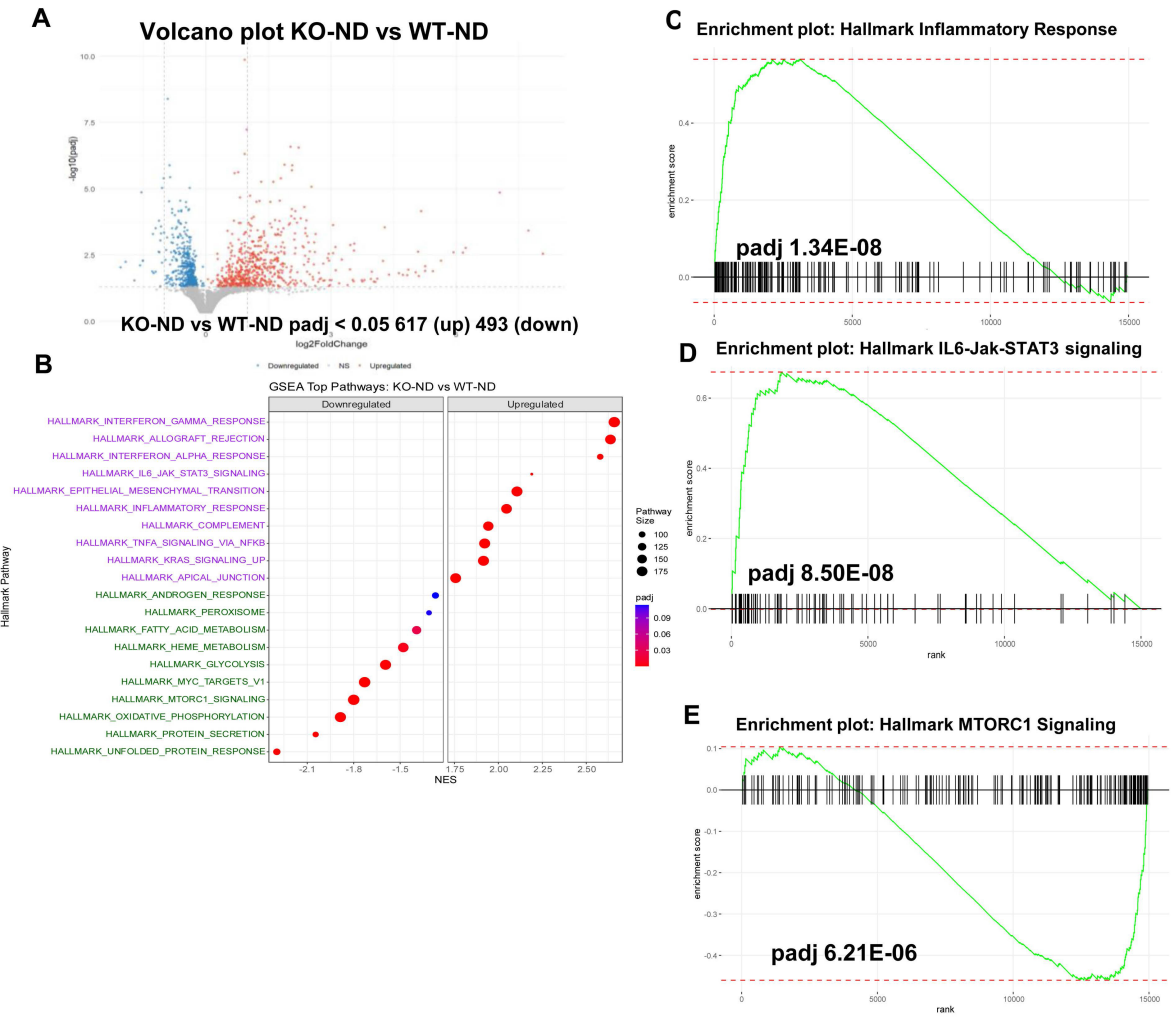
RNAseq analysis: KO-ND vs wt-ND. Total RNA was extracted from the pancreas of wt and Hpa2-KO mice fed normal diet (ND) and subjected to RNAseq analysis. Shown are the volcano plot (A) and the top GSEA pathways (**B**). GSEA pathway analyses of hallmarks of inflammatory response, IL6-Jak-STAT3, and MTORC1 are shown in C-E, respectively.

**Figure 8:**
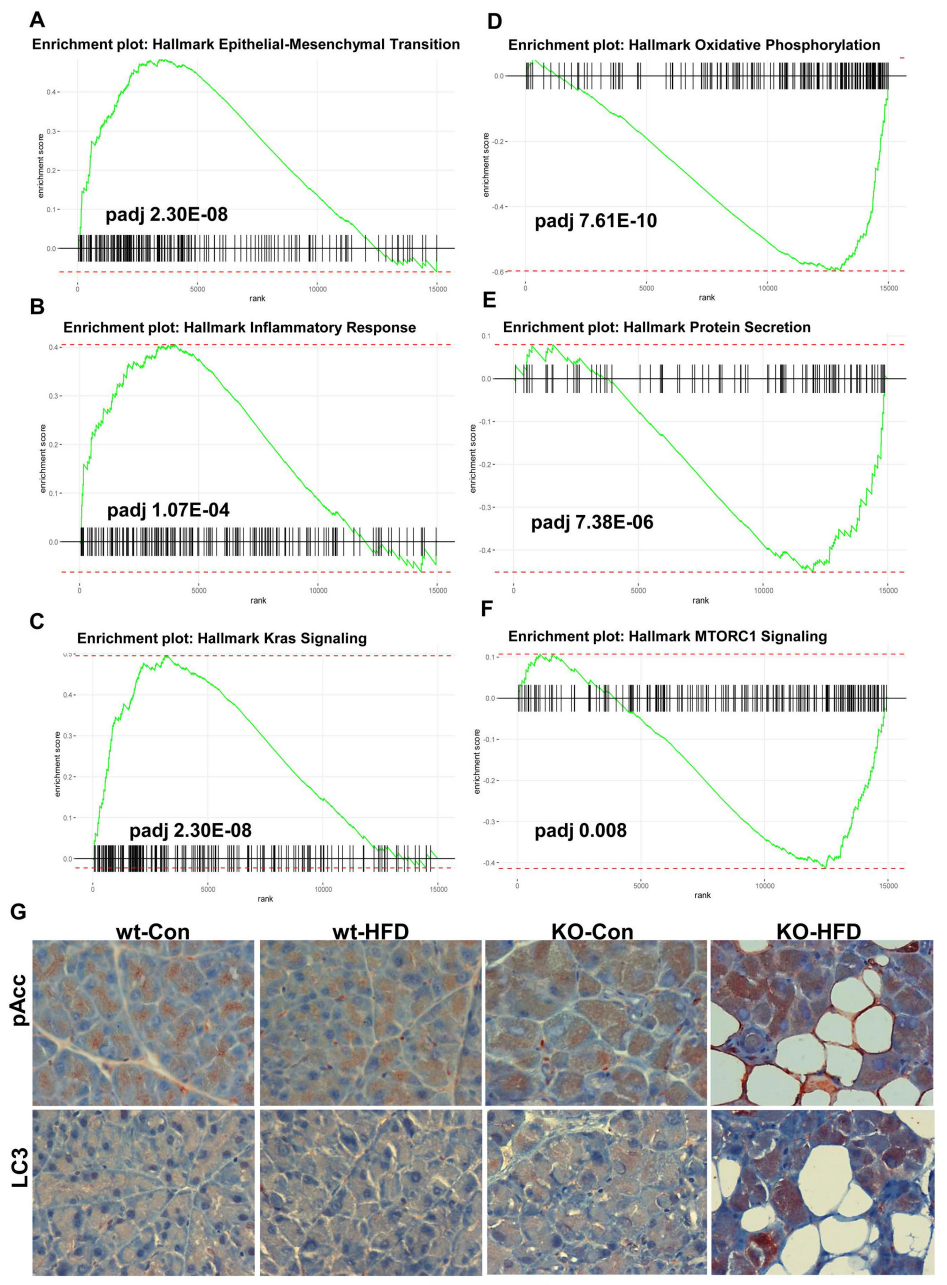
RNAseq analysis: KO-HFD vs KO-ND. Total RNA was extracted from the pancreas of Hpa2-KO mice fed normal diet (KO-ND) or HFD (KO-HFD) and subjected to RNAseq analysis. **A-F**. GSEA analyses. Shown are GSEA analyses of hallmark of epithelial-mesenchymal transition (A), inflammatory response (B), Kras signalling (C), oxidative phosphorylation (D), protein secretion (E), and MTORC1 signalling (F). **G**. Immunostaining. 5-micron sections of the indicated pancreas tissue were subjected to immunostaining applying antibodies directed against phospho-Acc (upper panels) and cleaved LC3 (lower panels). Shown are representative images at original magnification of x100.

We next performed transcriptomic analysis (RNAseq) on RNA extracted from the pancreas of wt and Hpa2-KO mice fed with HFD for 2 days. We first compared the control groups, WT-normal diet (ND) vs KO-ND, and found that the expression of many genes is altered in Hpa2-deficient pancreas (Volcano plot; Fig. 7A). GSEA analyses further revealed increased hallmark of inflammatory response in KO-ND vs WT-ND pancreas (Fig. 7B, C), in agreement with our current (Fig. 5) and previous results 15, along with increased hallmarks of IL6-Jak-STAT3 (Fig. 7D) and interferon ɣ response (Supplementary Fig. 5C) pathways. In contrast, the unfolded protein response was decreased substantially in KO-ND vs WT-ND (Supplementary Fig. 5D), and this was supported by decreased levels of Bip, a central component of the ER stress pathway (Supplementary Fig. 5E, F). Notably, the hallmark of the mechanistic target of rapamycin complex 1 (MTORC1) pathway was found to be decreased in KO-ND vs WT-ND (Fig. 7E). These results critically support the notion that Hpa2-deficiency results in molecular alterations that may prime the seemingly close-to-normal Hpa2-KO pancreas to external stimuli and conditions of stress. We next compared Hpa2-KO mice fed HFD for 2 days (KO-HFD) vs Hpa2-KO mice fed normal diet (KO-ND). At the top of GSEA pathways that were increased in KO-HFD was the hallmark of EMT (Fig. 8A), most likely representing the extensive ADM response in KO-HFD pancreas (i.e., Figs. 1, Supplementary Fig. 2-4). GSEA further indicated increased hallmark of inflammatory response in KO-HFD vs KO-ND (Fig. 8B), thus confirming the increased inflammation in Hpa2-KO pancreas following feeding the mice with HFD vs KO-ND (i.e., Fig. 5). Furthermore, the analysis shows increased expression of Kras signalling pathway that is strongly implicated in pancreatic cancer (Fig. 8C). Among the pathways that are found to be decreased were the hallmark of oxidative phosphorylation (Fig. 8D) and hallmark of protein secretion (Fig. 8E). Importantly, the analysis shows further decrease of MTORC1 pathway (Fig. 8F), and this was associated with activation of AMP-activated protein kinase (AMPK), evident by increased phosphorylation of Acetyl-CoA carboxylase (pAcc), an AMPK substrate (Fig. 8G, upper panels), and increased autophagy in KO-HFD pancreas (Fig. 8G, lower panels). These results position MTORC1 as a central hub that connects the key alterations elicited in response to Hpa2 deficiency. Thus, Hpa2 seemingly functions to maintain proper mTOR activity.

**Figure 9:**
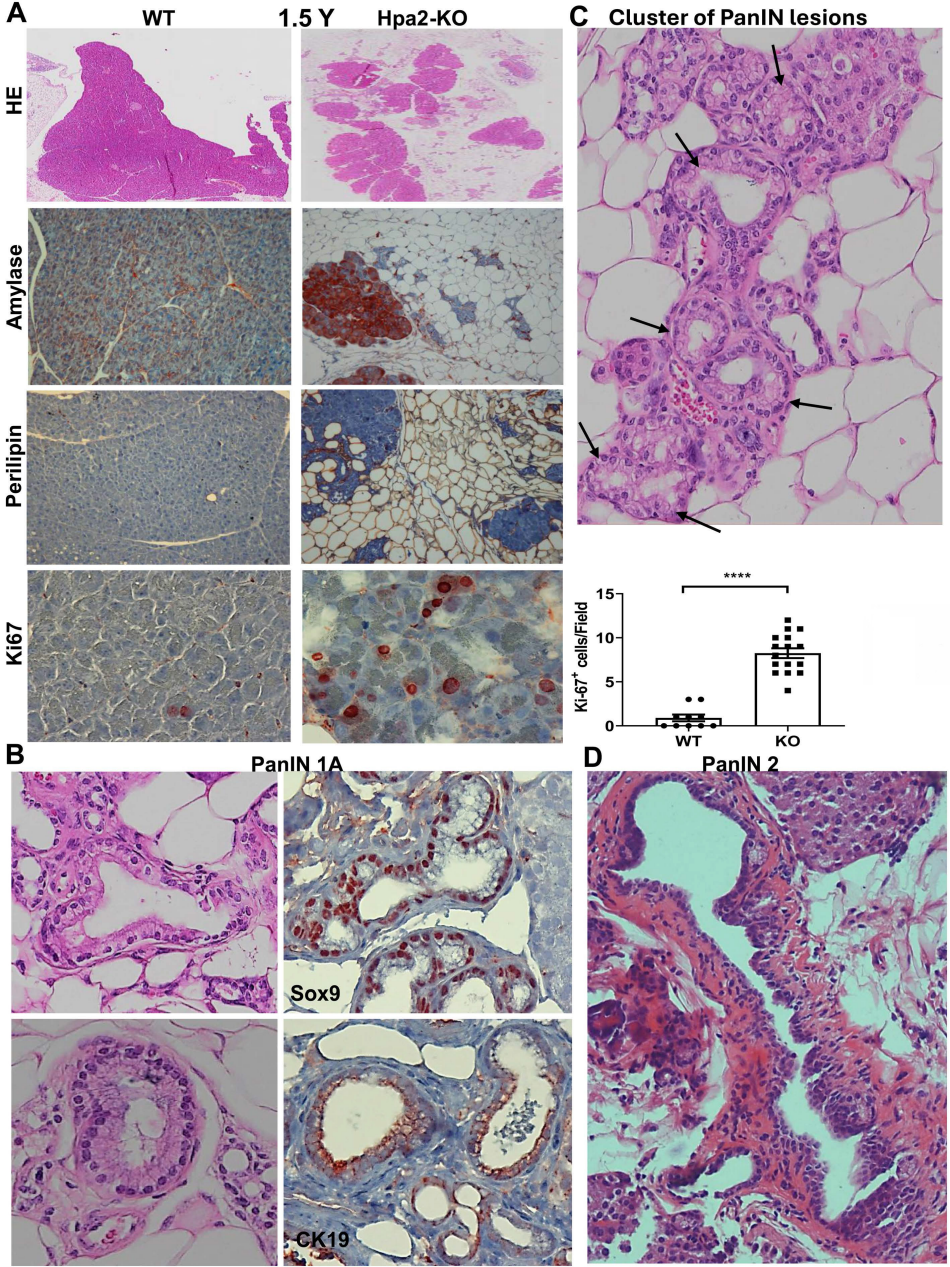
Aged Hpa2-KO mice develop PanIN lesions. Female wt and Hpa2-KO mice were left to age for 18 months (1.5 years). Mice were then sacrificed; pancreatic tissues were collected and fixed in formalin for histological examination. **A.** fatty pancreas. 5-micron sections were stained for hematoxylin and eosin (HE; upper panels) or were subjected to immunostaining applying antibodies directed against alpha-amylase (second panels), perilipin-1 (third panels), and Ki67 (lower panels). Quantification of Ki67-positive acinar cells is shown graphically in the lower-right panel. **B, C**. PanIN. Examination of the HE stains at high magnification readily identified PanIN 1A lesions only in Hpa2-KO pancreas (**B**, left panels) that are stained positively for Sox9 and cytokeratin (CK)19 (**B**, right panels). PanIN 1A lesions in Hpa2-KO pancreas are often organized in clusters (**C**; black arrows), surrounded by fat cells. **D**. Prolonged HFD. Hpa2-KO female mice were fed with HFD for 8 months. Mice were then sacrificed, and the pancreas was subjected to histological examination. Shown is a representative PanIN 2 lesion (original magnification x 100).

**PanIN develops spontaneously in the pancreas of aged Hpa2-KO mice**. Unlike Hpa2-KO male mice, Hpa2-KO female mice develop ADM and fatty pancreas spontaneously, evident already 6 weeks following the administration of tamoxifen 15. Given that these alterations are considered pro-tumorigenic 40-42, we next examined conditions that will advance ADM to neoplasia. We first examined the pancreas of aged (1.5-year-old) Hpa2-KO female mice histologically. We found that the fatty pancreas of young (i.e., 3-month-old 15) Hpa2-KO mice was not recovered with age, and aged mice exhibited comparable or even greater content of fat cells (Fig. 9A). This emerges from histological examination (Fig. 9, upper panels) and immunostaining for alpha-amylase (Fig. 9A, second panels), perilipin (Fig. 9A, third panels), and Ki67 (Fig. 9A, lower panels). Altogether, these results suggest that the fatty pancreas phenotype of Hpa2-KO female mice is not restored with time. Pathological examination at high magnification readily identified PanIN 1A lesions (Fig. 9B), often organized in clusters (Fig. 9C), only in aged Hpa2-KO female pancreas. Encouraged by this result, we next fed Hpa2-KO female mice with HFD for extended periods. Histological examination of the pancreas after 8 months of HFD feeding identified papillary duct lesions with atypia that were classified as more advanced, PanIN 2, lesions (Fig. 9D). This implies that time itself is sufficient to advance the formation of neoplasia in Hpa2-KO, but not WT pancreas, while time and HFD result in even more advanced lesions.

## Discussion

The presence of fat cells within the pancreas has been recognized for over a century through imaging studies performed for other indications; it was considered an incidental finding, and its clinical implications were not thoroughly investigated for several decades 41, 43. The development of advanced imaging techniques revolutionized our understanding of the significance of fat accumulation in pancreatic biology 41. These advanced technologies led to the estimation that fatty pancreas disease (FPD) is more frequent than type 2 diabetes mellitus and acute pancreatitis, combined 41. Moreover, accumulating evidence gathered in recent years supports the association of fatty pancreas with the development of pancreatic cancer as well as other pathologies of the human pancreas 41, 43-48. Indeed, some pharmacological interventions, including glucagon-like peptide-1 (GLP-1) receptor agonists and sodium-glucose cotransporter-2 (SGLT2) inhibitors, have shown potential to reduce pancreatic fat 46.

However, the mechanisms responsible for fatty pancreas are not fully understood, and more research is required to better define the molecular and cellular mechanisms underlying this phenomenon to advance early detection and therapeutic intervention. The results presented here highlight Hpa2 as a key player that protects the pancreas against FPD. The pronounced effect of HFD on the pancreas of Hpa2-KO mice was unexpected and unique, as the extensive literature on this model typically reports effects only on the liver under various HFD regimens. While the liver of Hpa2-KO mice was affected by HFD, the severity of liver pathology appeared similar in wt-HFD and KO-HFD mice. The basis for this organ specificity remains to be fully defined. It should be noted that pancreatic acinar cells are considered the most metabolically active cells in the body because they continuously synthesize, fold, store, and secrete large quantities of digestive enzymes 49. Most likely, acinar cells of the Hpa2-KO pancreas are particularly vulnerable to metabolic and inflammatory stress induced by high-fat diet, and even modest alterations may shift the balance toward metaplasia and early neoplastic transformation. In contrast, metabolically adaptive organs such as the liver may better tolerate metabolic stress conditions and alterations in heparan sulfate dynamics under similar conditions. Alterations in the histology and function of other organs and tissues in the Hpa2-KO mouse following HFD have yet to be explored. Previously, we found that the development of fatty pancreas in Hpa2-KO mice varies by gender: it develops spontaneously in female mice as early as 6 weeks after tamoxifen administration (to knock out the Hpa2 gene), whereas the Hpa2-KO male pancreas retains a relatively preserved morphology but appears inflamed 15. Notably, male Hpa2-KO mice responded intensely to cerulein and developed fatty pancreas within one day of treatment 15, suggesting that while the pancreas morphology is relatively preserved, an altered molecular mechanism(s) was already established, and was fully activated by this short stimulation. Here, we revealed HFD as a novel condition that rapidly (within 3 days) and efficiently stimulates the formation of fatty pancreas in Hpa2-KO male mice. The molecular mechanism(s) linking these three conditions (female vs male, cerulein treatment, HFD) and resulting in fatty pancreas in Hpa2-KO mice are presently unclear but may involve mTOR (see below).

In fact, HFD resulted not only in fatty pancreas but also in extensive ADM and increased inflammation. These phenotypes (fatty pancreas, ADM, inflammation) are likely interconnected and affect one another in many ways. For example, fat cells in the pancreatic parenchyma can lead to chronic inflammation because adipocytes produce proinflammatory cytokines, chemokines, and chemoattractants 50 that promote the massive recruitment of immune cells. The accumulation of B-cells in the Hpa2-KO pancreas after 13 weeks of HFD clearly illustrates a chronic phase of inflammation. In addition, given that the Hpa2-KO male pancreas is already inflamed (Fig. 5) 15, activation of existing immune cells by HFD can amplify inflammation, as was found in the Hpa2-KO pancreas following HFD. Increased inflammation can stimulate ADM, as often observed in pancreatitis 40, 51, 52. More specifically, macrophages are present in untreated Hpa2-KO male pancreas and increased in number in mice fed with HFD. Macrophages were noted to elicit ADM during acute pancreatitis, mediated by the secretion of cytokines and by enhancing ECM deposition 40, 51-54. Moreover, macrophages can be activated by various metabolic signals, including free fatty acids. This might lead to fibrotic replacement of the acinar cells 50, as was noted in Hpa2-KO pancreas following HFD regimens, evident by increased alcian blue, Masson's Trichrome, and SMA staining. Increased macrophage abundance in Hpa2-KO pancreas is evident as early as 3 days on HFD, likely contributing to stress conditions that elicit ADM. This creates a unique fibrotic and inflamed microenvironment, typical of pancreatic tumors, already at a pre-neoplastic stage. Notably, macrophages isolated from Hpa2-KO mice are shifted towards M2 phenotype and exhibit pro-tumorigenic properties 16. Importantly, M2 macrophages have been shown to drive ADM 55. In pancreatic cancer, increased abundance of M2 macrophages was found to orchestrate an immunosuppressive microenvironment and promote the progression of PanIN to PDAC 51, mediated in part by interleukin-1 receptor antagonist (IL-1ra), which is increased in KO-HFD pancreas (Supplementary Table 2). It should be nonetheless noted that both inflammatory (M1) and alternatively activated (M2) macrophages induce ADM and synergize to drive lesion growth 51. Thus, Hpa2 deficiency results in pancreatic microenvironment that is pro-tumorigenic (also see below). Given that macrophages are already present in the control, untreated Hpa2-KO male pancreas, it is possible that these cells trigger the response to HFD; however, further studies are required to confirm this.

ADM is considered a form of epithelial-to-mesenchymal transition (EMT) thought to play an important role in tumor growth and metastasis 56. Accumulation of adipocytes, possibly due to acinar to adipocyte trans differentiation (AAT) 15, is also considered a form of EMT 57, suggesting that acinar cells in Hpa2-KO pancreas lost their differentiation programme and exposed their high capacity for intrinsic cell plasticity 56. Importantly, we found that the levels of GATA4, GATA6, MIST, and PDX1, pancreatic transcription factors that play a critical role in acinar cell differentiation and maturation 37, 42, 58-61, are profoundly decreased in the KO-HFD pancreas. Notably, GATA4 and GATA6 share partially redundant functions during pancreas organogenesis, and inactivation of both GATA4 and GATA6 results in pancreas agenesis, whereas inactivation of each transcription factor alone does not affect pancreas formation 37. Reduced levels of GATA4 and GATA6, along with reduced levels of MIST1 and PDX1 in the Hpa2-KO pancreas following HFD, thus result in acinar cell de-differentiation and EMT, leading to ADM and fatty pancreas 42. Moreover, the acinar differentiation program acts as a tumor suppressor in the pancreas, and low expression of GATA6 and GATA4 was associated with worse outcomes of PDAC patients, and with liver metastasis 37.

ADM is a natural reversible mechanism that becomes irreversible when acinar cells acquire Kras mutation or are under continuous stress 42, 62. The remarkable plasticity of acinar cells and the formation of ADM are essential for their regenerative capacity following pancreatic injury, but also render them susceptible to trans-differentiation into PanIN upon exposure to oncogenic stress, which can further lead to PDAC 62. We found that ADM progresses spontaneously to pre-malignant PanIN 1A in aged Hpa2-KO pancreas, and that even more advanced PanIN 2 lesions developed in Hpa2-KO mice fed with HFD for 8 months. These results clearly show that Hpa2 function protects acinar cells from oncogenic transformation and pancreatic neoplasia. Further progression into PDAC typically requires active, most often mutated (i.e., G12D) Kras. Breeding of our Hpa2-KO mice with Kras+/LSLG12D; Ptf1a-CreER mice to bring active Kras into the Hpa2-null pancreas is currently ongoing.

The significance of these results emerges from the finding that cells may disseminate from such early, low-grade PanIN lesions into the bloodstream at a time when no primary tumor is detected in the pancreas 42. Thus, low Hpa2 levels may indicate that patients are at high risk of developing aggressive PDAC, and quantification of Hpa2 levels may be considered a routine diagnostic parameter. Of note, while Hpa2 levels are markedly reduced in conditions of sepsis, therapeutic plasma exchange (TPE) significantly improves hemodynamic instability in a mouse model of sepsis, likely restoring Hpa2 levels to near-normal levels 29. Employing Hpa2-based therapy in individuals consuming HFD and exhibiting low expression levels of Hpa2, in cancer patients, and patients under acute or chronic inflammation (i.e., pancreatitis) awaits further investigation and identification of therapeutic Hpa2-derived domains and peptides. Given that acute pancreatitis (AP) is the third leading cause of hospital admission due to digestive disease in many Western countries 63, the clinical potential of new therapeutic modalities in AP and other diseases of the pancreas is very important.

The molecular mechanism underlying the severe histological and functional alterations in the Hpa2-KO pancreas likely involves MTORC1, and key findings in Hpa2-deficient pancreas can be explained by decreased MTORC1 activity. MTORC1 is a master regulator of cell growth, protein synthesis, metabolism, and nutrient sensing. Dysregulation of mTOR signalling is associated with various diseases such as obesity, cancer, and neurological disorders 64. In the pancreas, MTORC has mainly been studied in the context of β-islets, revealing that mTOR has both positive and negative effects on pancreatic β-cells in the development of diabetes 65. Our results suggest that MTORC1 is also critically important in the exocrine pancreas and is regulated by Hpa2. In the absence of Hpa2, MTORC1 activity is decreased, accompanied by severe morphological alterations in the pancreas. The first layer, and possibly the most important, is the reduced levels of key transcription factors responsible for acinar cell differentiation (i.e., GATA4, GATA6, MIST). Transcriptomic studies of ADM models have reported that during the early stages of acinar reprogramming, MTORC1 is downregulated, associated with the loss of acinar markers and the induction of progenitor/metaplastic programs (e.g., SOX9; 66). Thus, while active MTORC1 is linked to maintenance of the differentiated phenotype under homeostasis, its suppression is part of the reprogramming process toward ADM 66. This suggests that lower MTORC1 is part of the cellular transition to a less differentiated, more plastic state 66. This critically unravels the extensive ADM and AAT in the KO-HFD pancreas. Notably, decreased MTORC1 is also evident in control Hpa2-KO pancreas under normal diet (KO-ND) vs WT-ND, without a noticeable phenotype. However, upon feeding with HFD, AMPK, which functions to inhibit MTORC1 67, is activated, resulting in further attenuation of MTORC1 and extensive ADM. Reduced metabolism of serine, glycine, and threonine, as well as metabolism of cysteine and methionine, can signal a nutrient-poor state, even in the presence of overall nutrient excess from HFD, and contribute to MTORC1 suppression. Consequently, the block of autophagy by active MTORC1 is decreased 68, allowing increased autophagy. Notably, mTORC1 is not only a driver of anabolic growth and protein synthesis, but also a regulator of mitochondrial activity, biogenesis, and oxidative phosphorylation 69. Decreased hallmark of oxidative phosphorylation in KO-HFD vs KO-ND (Fig. 8D), thus, agrees with reduced MTORC1 activity. Furthermore, decreased levels of many mitochondrial ribosome protein subunits (Supplementary Table 2), protein secretion, and glycine, serine, and threonine metabolism are all seemingly related to decreased MTORC1 activity 70. This implies that Hpa2, through the regulation of MTORC1, is intimately engaged in ribosomal and mitochondrial functions. Studies also show that inhibiting mTORC1 activity can exacerbate inflammation, particularly under conditions of high-fat diet and metabolic stress. More specifically, mTOR is implicated in macrophage function 71, 72, which seems more abundant in KO-HFD vs KO-ND pancreas.

Transformation of acinar cells to duct-like structures [acinar to ductal metaplasia (ADM)] and into adipocytes [acinar to adipocyte transdifferentiation (AAT)] is thought to be the leading mechanism responsible for the histological alterations of the Hpa2-KO pancreas following HFD, the result of inherent plasticity of differentiated acinar cells. This conclusion was based on genetic lineage tracing and other methodologies following the silencing of acinar transcription factors, including GATA6, MIST1, and c-Myc 57, 59. Alternatively, several studies have proposed that multipotent biliary tree stem/progenitor cells located within peribiliary glands (PBGs) of the extrahepatic bile ducts can give rise to pancreatic-committed progenitors located in pancreatic duct glands (PDGs) within the intrapancreatic ductal system 73-75. In this model, a proximal-to-distal maturation axis extends from PBGs to PDGs, with progressive commitment toward pancreatic endocrine and exocrine lineages 75, 76. Notably, PDGs are considered a potential niche for PanIN initiation, and can support the formation of pre-cancerous pathology 74, and biliary cancer stem cells were identified in patient-derived tissue 73. However, definitive in vivo lineage-tracing evidence supporting a significant contribution of this system to adult pancreatic acinar or endocrine maintenance remains limited 77. The possible contribution of such cells to ADM, PanIN 1 that develops in aged Hpa2-KO pancreas, and/or PanIN 2 that develops in Hpa2-KO pancreas following HFD is yet to be resolved.

Taken together, our results robustly and systemically identify HFD as a condition that elicits extensive morphological and functional alterations in Hpa2-deficient pancreas and identify MTORC1 as a critical hub that orchestrates Hpa2 function. Unlike cerulein, which was used in our previous study, HFD represents a most eminent and common physiological condition that increases the risk of developing several pathologies, including pancreatitis, type 2 diabetes mellitus (T2DM), and PDAC. Interestingly, despite extensive literature on mTOR, its role in pancreatic diseases beyond diabetes and PDAC remains poorly explored. The results of this study may open new directions in the study and treatment of fatty pancreas and pancreatitis.

## Supplementary Material

Supplementary figures and tables.

## Data Availability

All data generated and analyzed in this study are presented in this published article and its supplementary files. All primary data can be made available on request from the corresponding authors.

## Abbreviations

AAT: acinar-to-adipocytes trans-differentiation
ADM: acinar to ductal metaplasia
AP: acute pancreatitis
DEG: differentially expressed genes
ECM: extracellular matrix
FPD: fatty pancreas disease
GSEA: gene set enrichment analysis
HFD: high-fat diet
HS: heparan sulfate
HSPG: heparan sulfate proteoglycan
KO: knockout
MTORC1: mechanistic target of rapamycin complex 1
ND: normal diet
PanIN: pancreatic intraepithelial neoplasia
TPE: therapeutic plasma exchange
WT: wild type
