# Supplementary material for: Heparanase 2 (Hpa2) protects the exocrine pancreas from damages of Western diet and pancreatic cancer
Source: Int J Biol Sci. 2026 May 1;22(10):4997–5014. doi: 10.7150/ijbs.131032 (PMC13215069; doi:10.7150/ijbs.131032)
Supplement: Supplementary file 1 — Supplementary figures and tables. [file ijbsv22p4997s1.pdf]

Suppl. Figure legends

Suppl. Figure 1. Liver analyses. WT and Hpa2-KO mice were fed regular mouse chow as control (Con) or HFD for 13 weeks. **A, B**. Body weight. Mice were weighed once a week, and the collective measurements are shown in (**A**); average mouse weight at termination (13 weeks) is shown in (**B**). **D,E**. Biochemical analyses. Mice were then anesthetized, blood was collected, and the levels of ALT (**D**) and cholesterol (**E**) were quantified by routine biochemical hospital laboratory. Mice were then sacrificed, livers were collected, weighed (**C**), fixed in formalin, and subjected to histological examinations. Shown are representative H&E (**F**, upper panels) and Sirius red (**F**, lower panels) staining. Original magnifications: x10.

Suppl. Figure 2. Accumulation of fat cells (fatty pancreas), inflammatory cells, and ADM structures in Hpa2-KO pancreas after 8 weeks of HFD. WT and Hpa2-KO mice were fed regular mouse chow as control (Con) or HFD for 13 weeks. Mice were then sacrificed, and pancreatic tissues were collected and fixed in formalin for histological examinations. Shown are representative images of hematoxylin & eosin (HE) at low (x2.5; **A**, upper panels) magnification. Acinar to ductal metaplasia (ADM) and foci of inflammation are shown at high (x100) magnification (second and third right panels). Enlarged beta islets are shown in the lower right panel. **B**. Body weight. Mice were weighed once a week, and the collective measurements are shown graphically. Mice were then sacrificed; pancreas tissues were collected and weighed (**C**); pancreas-to-body weight values are presented in (**D**). Average beta islet areas in each group are shown graphically in (**E**).

Suppl. Figure 3. Accumulation of fat cells (fatty pancreas), inflammatory cells, and ADM structures in Hpa2-KO pancreas after 4 weeks of HFD. WT and Hpa2-KO mice were fed regular mouse chow as control (Con) or HFD for 4 weeks. Mice were then sacrificed, and pancreas tissues were collected and fixed in formalin for histological examinations. Shown are representative images of hematoxylin & eosin (HE) at low (x2.5; **A**, upper panels) magnification. Abundance of fat cells, multiple structures of acinar to ductal metaplasia (ADM), and foci of inflammation are shown at high (x100) magnification in the right panels, respectively (second, third, and fourth panels). **B, C**. Body weight. Mice were weighed once a week, and the collective measurements are shown graphically (**B**). Body weight at termination (4 weeks) is shown graphically in (**C**). Mice were then sacrificed; pancreas tissues were collected and weighed (**D**); pancreas-to-body weight values are presented in (**E**).

Suppl. Figure 4. Accumulation of fat cells (fatty pancreas), inflammatory cells, and ADM structures in Hpa2-KO pancreas after 1 week of HFD. WT and Hpa2-KO mice were fed regular mouse chow as control (Con) or HFD for 1 week. Mice were then sacrificed, and pancreas tissues were collected and fixed in formalin for histological examinations. Shown are representative images of hematoxylin & eosin (HE) at low (x2.5; **A**, upper panels) magnification. Abundance of fat cells, multiple structures of acinar to ductal metaplasia (ADM), and foci of inflammation are shown at high (x100) magnification in the right panels, respectively (second, third, and fourth panels). **B, C**. Body weight. Body weight at termination (1 week) is shown graphically in (**B**). Mice were then sacrificed; pancreas tissues were collected and weighed (**C**); pancreas-to-body weight values are presented in (**D**). Average beta islet areas in the experimental groups are shown graphically in (**E**).

Suppl. Figure 5. Densitometry analyses. The intensity of PPARɣ (upper panel) and cytokeratin 19 (CK19; lower panel) bands in the immunoblotting (Fig. 4C) was quantified by the ImageJ Pro software and is shown graphically in arbitrary units after normalization to the levels of ERK2. **C-D**. GSEA. Hallmark of interferon ɣ response is shown for KO-ND vs WT-ND (C); Hallmark of unfolded protein response is shown for KO-HFD vs KO-ND (D). **E**. Immunoblotting. Extracts of the indicated pancreas (each lane represents a pool of pancreas tissue extracted from 2-3 mice) were subjected to immunoblotting, applying antibodies directed against Bip (upper panel) and ERK2 (lower panel as loading control). Densitometry analysis of Bip is shown graphically in **F**.

Suppl. Figure 6. KEGG pathway analysis. Proteins were extracted from 10-micron paraffin sections of KO-HFD vs WT-HFD pancreas and were subjected to KEGG analysis. Shown are graphical representations of pathways that were increased (**A**) or decreased (**B**) in KO-HFD vs WT-HFD pancreas. Red arrows points to pathways that are most relevant to our results.

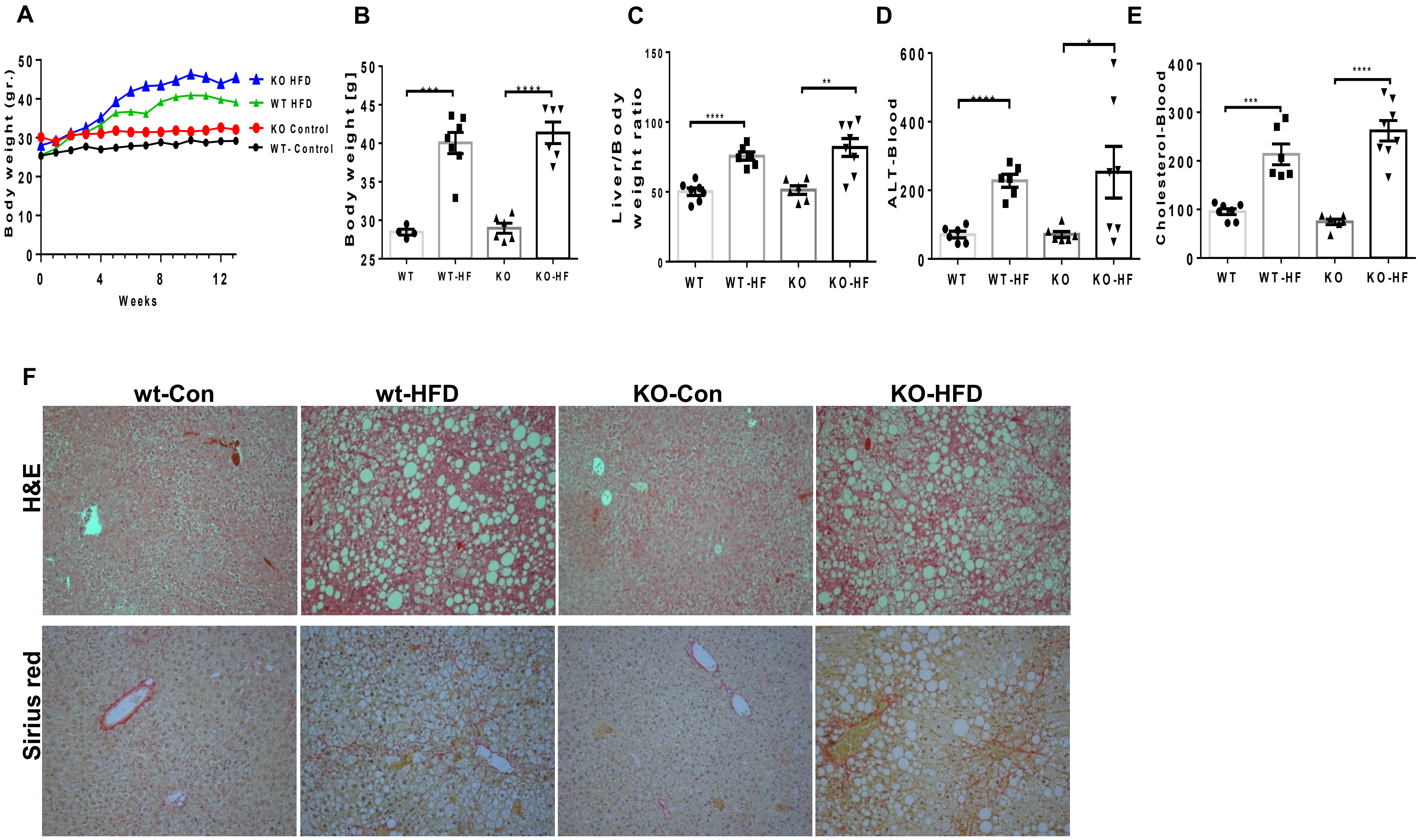

Suppl. Figure 1

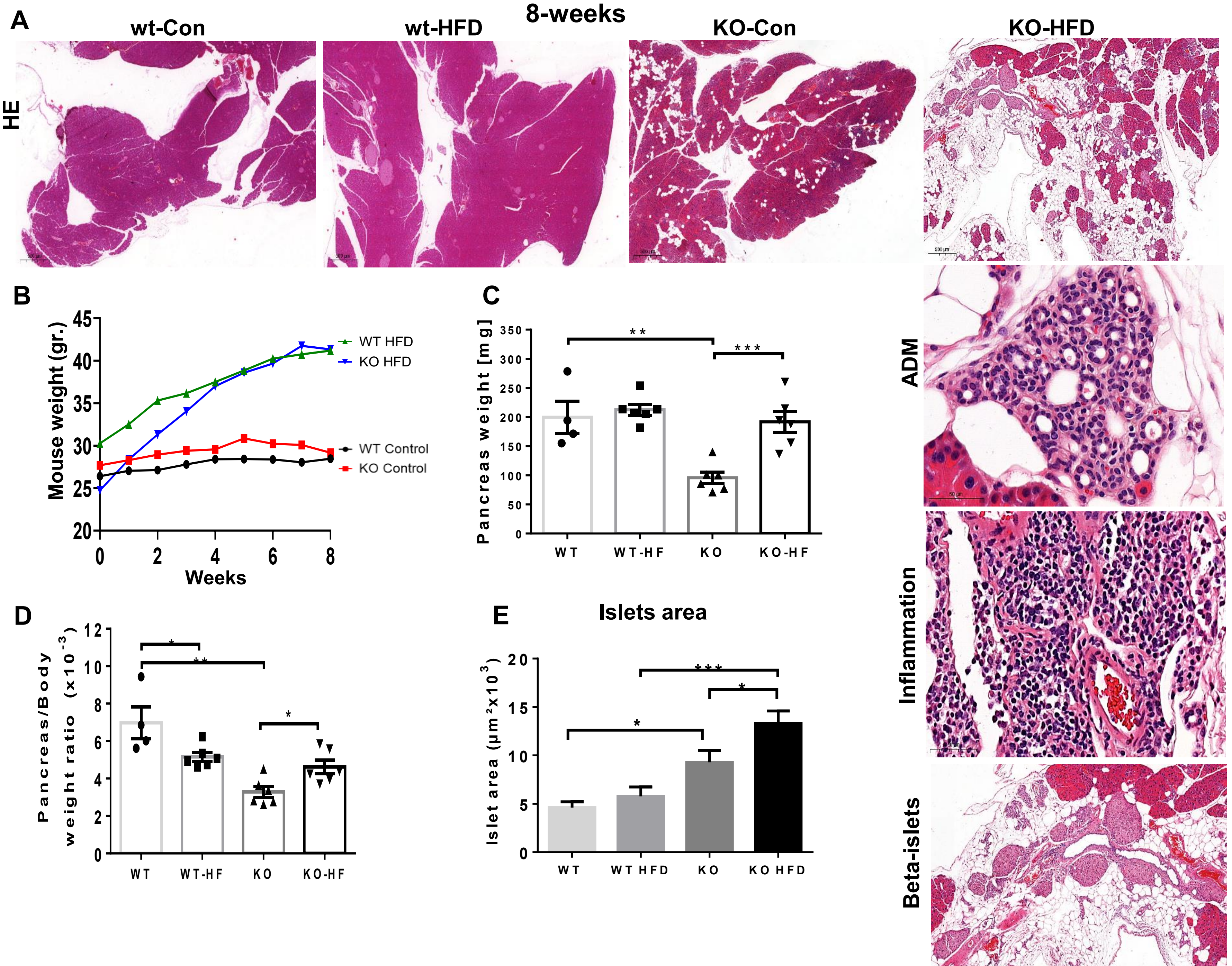

Suppl. Figure 2

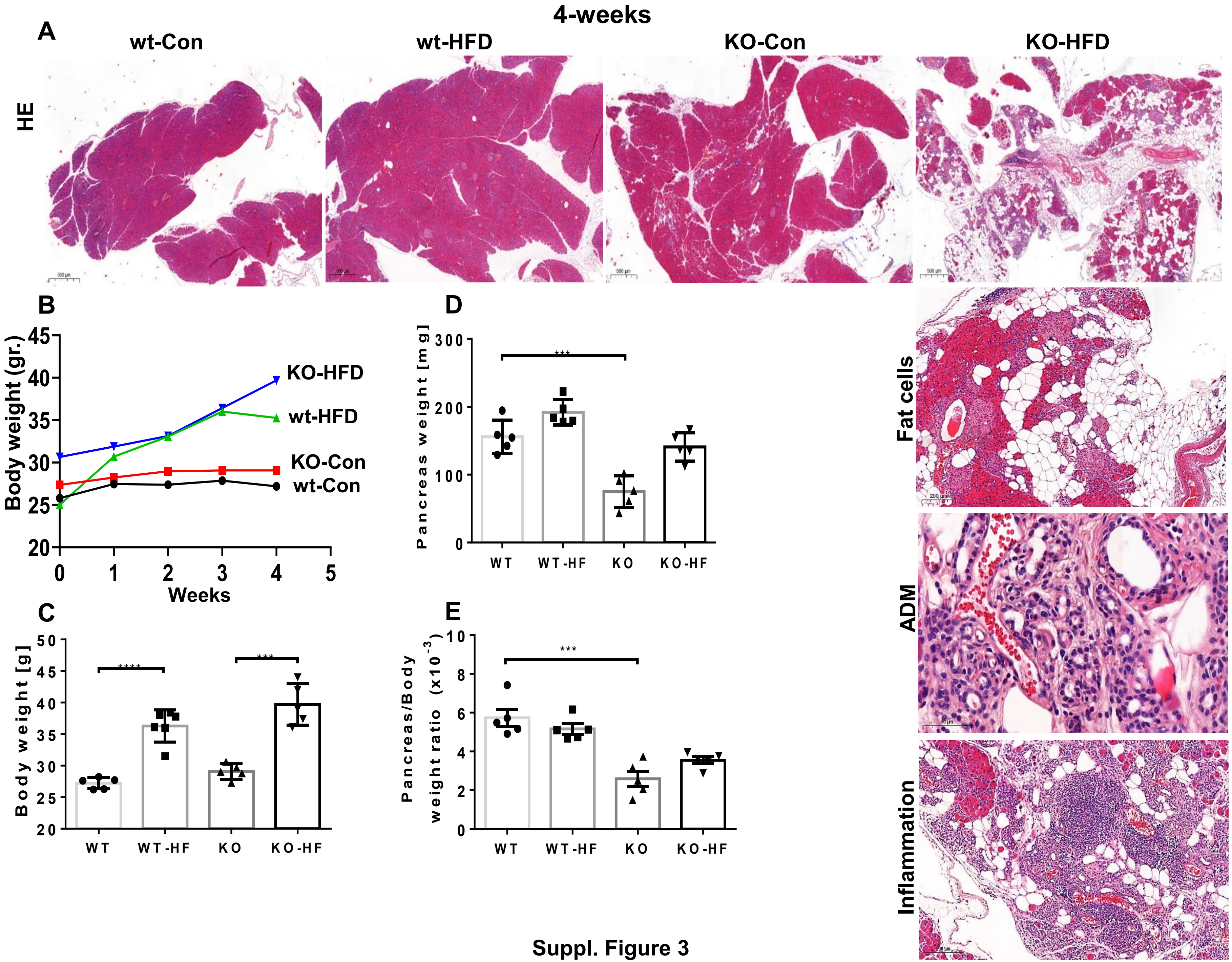

Suppl. Figure 3

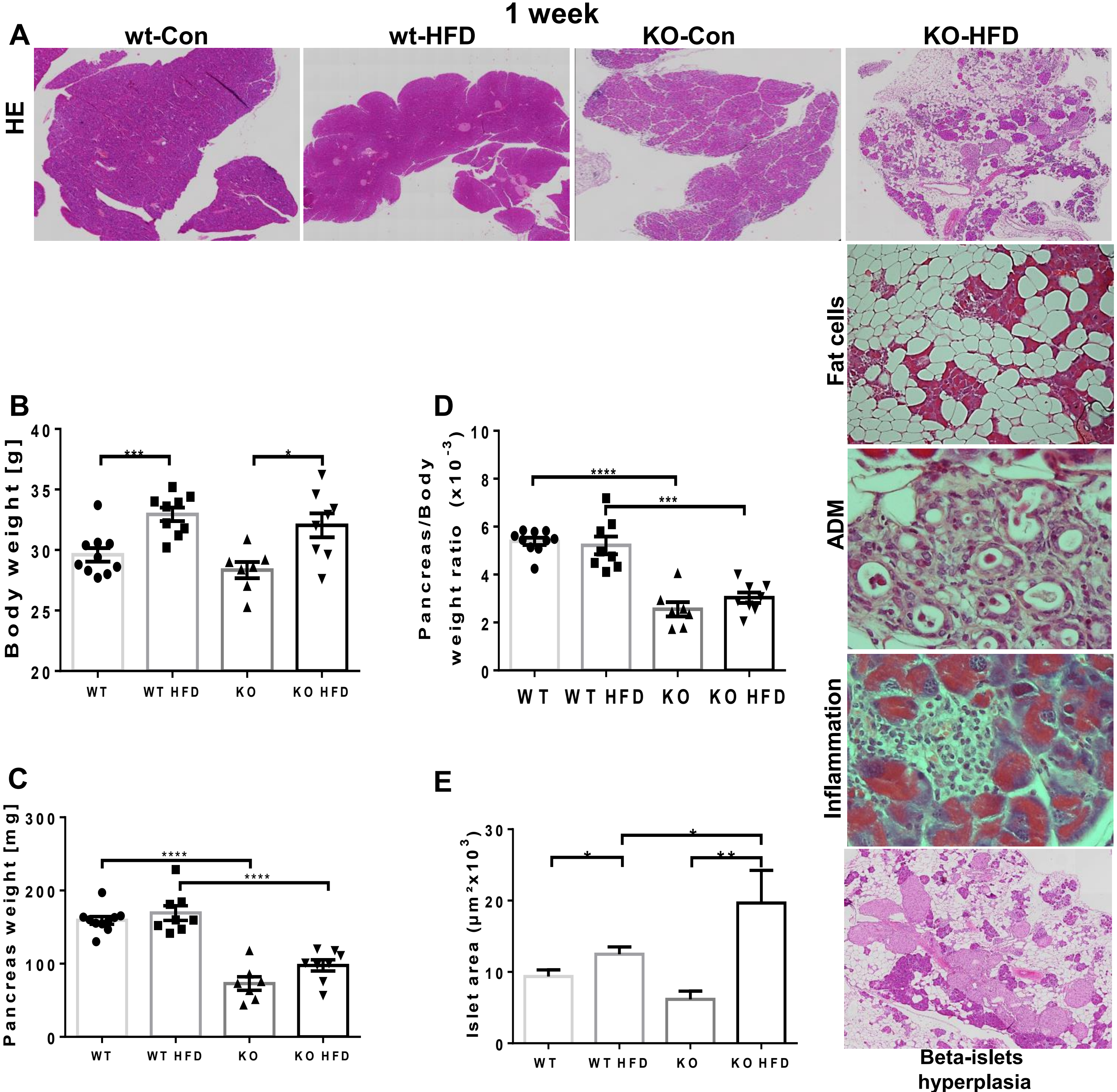

Suppl. Figure 4

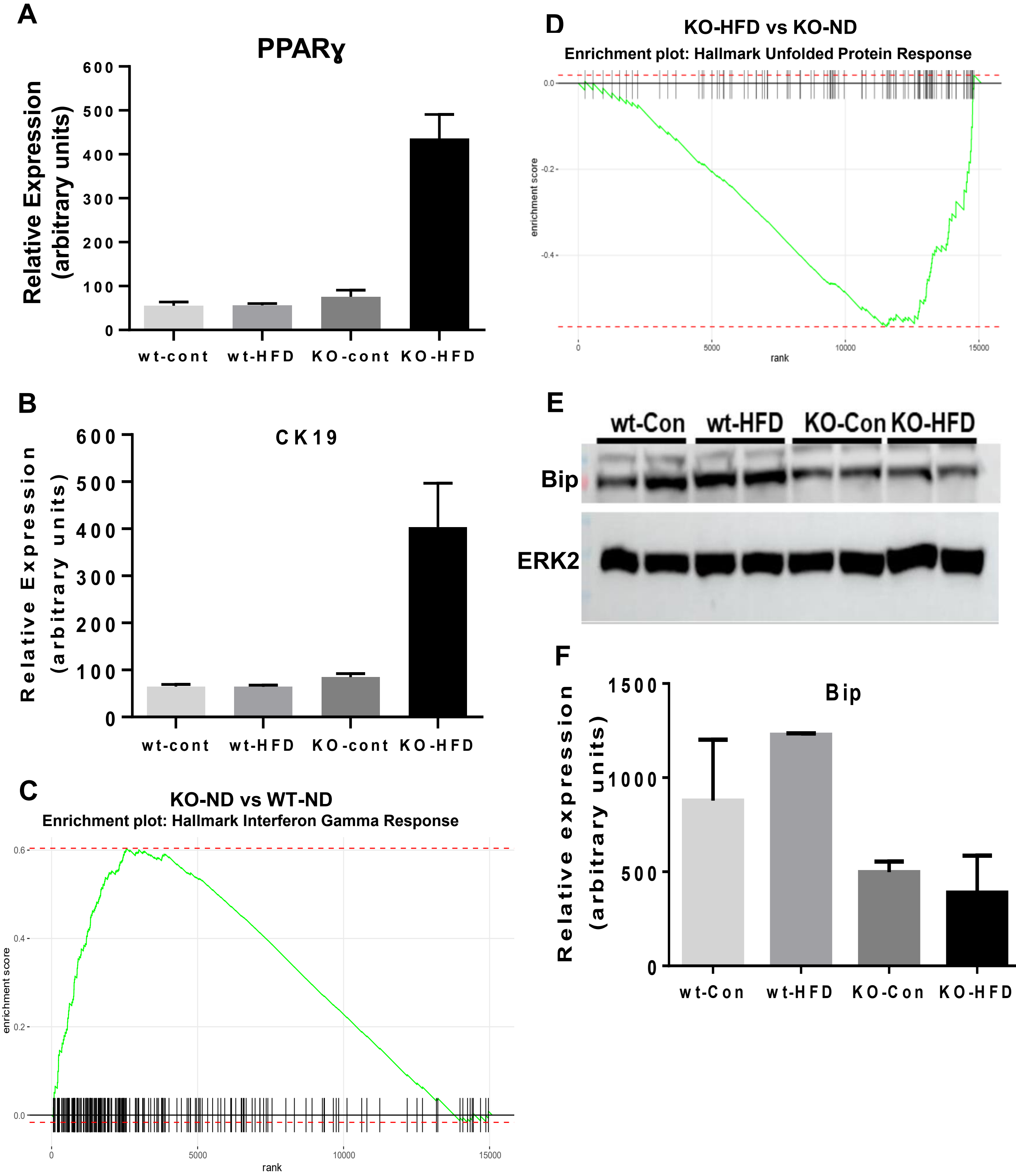

Suppl. Figure 5

Increased in KO-HFD vs WT-HFD

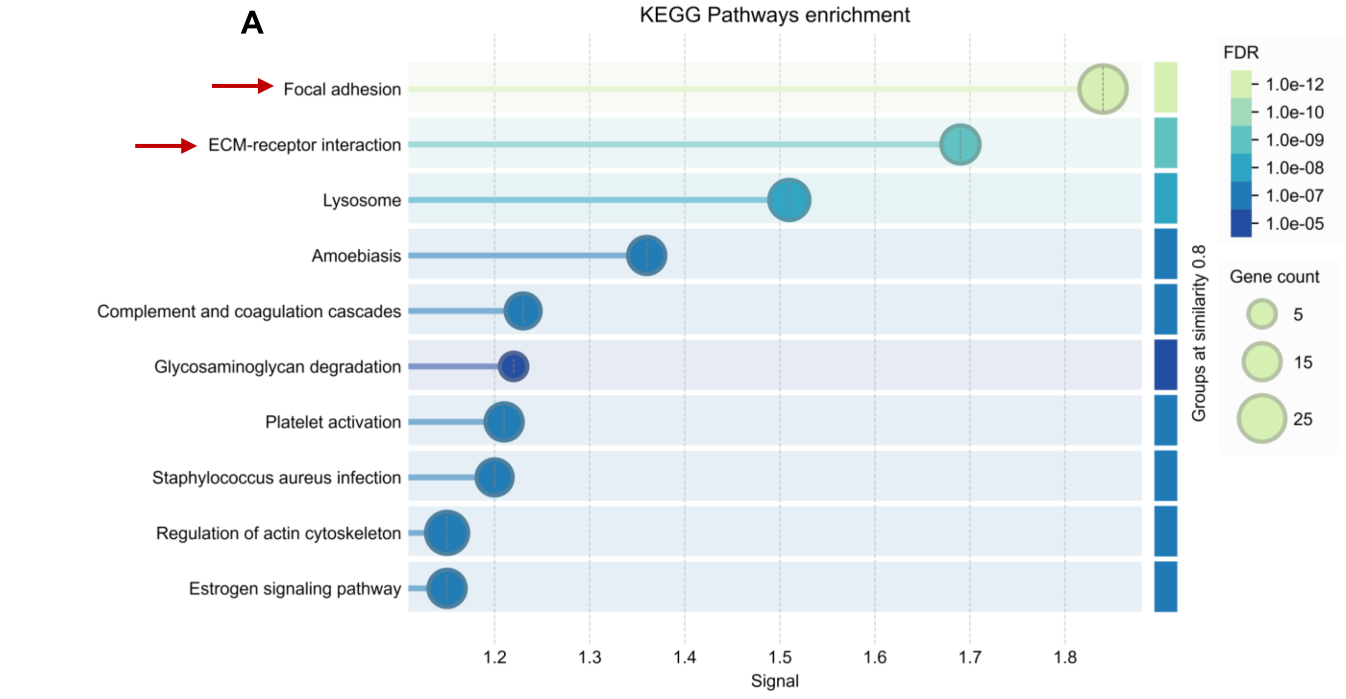

Decreased in KO-HFD vs WT-HFD

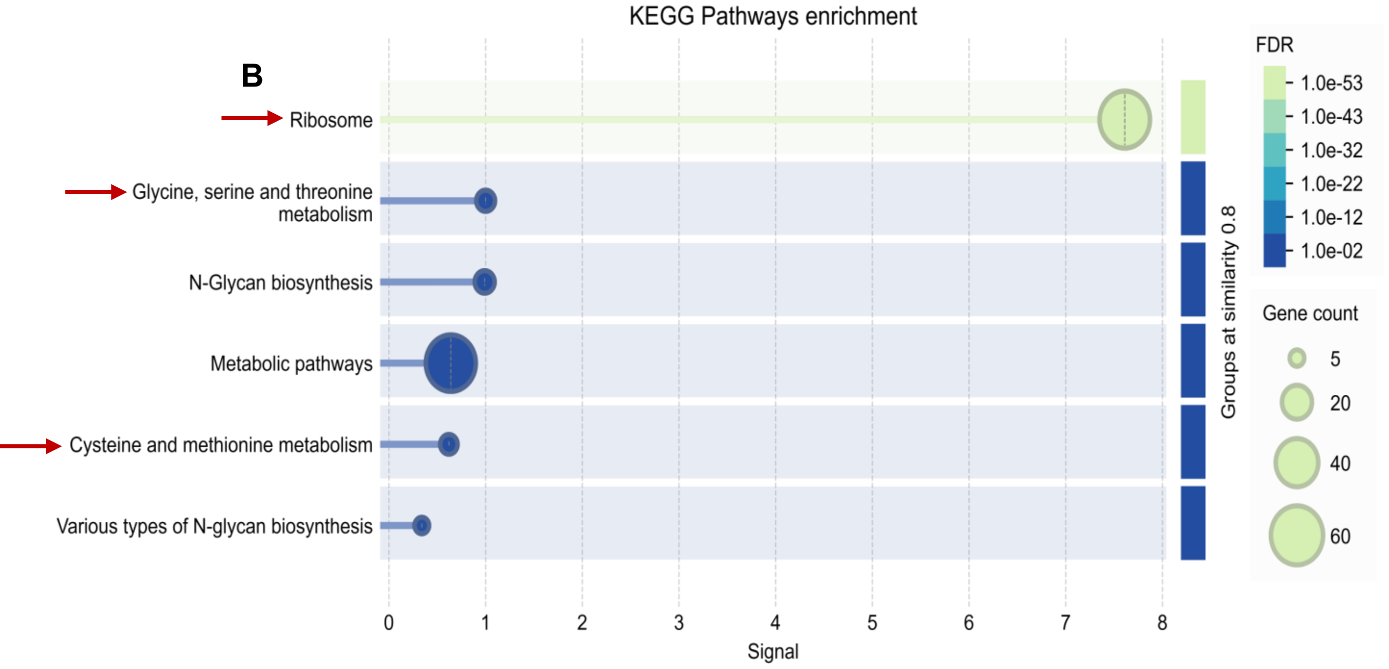

Suppl. Figure 6

Suppl. Table 1: Diet composition

Composition	Control diet, kcal/kg (% kcal from)	HFD, kcal/kg (% kcal from)
Fat	367 (11)	1890 (42)
Protein	768 (24)	684 (15.2)
Carbohydrate	2091 (65)	1921 (42.7)
**Nutritional values (g/kg)**	**Control diet**	**HFD**
Crude protein	192	195 (casein)
Carbohydrate	534	491 (corn starch and sucrose)
Polysaccharides	391	150 (starch)
Disaccharides	47.8	341 (sucrose)
Crude fat	40	212
Myristic acid C-14:0	-	23
Palmitic acid C-16:0	-	60.9
Stearic acid C-18:0	1.2	27.3
Oleic acid C-18:1	6.8	56.7

Suppl. Table 2. Changes of protein levels in KO-HFD vs KO-Con pancreas

Protein	Log2	Fold change	Protein	Log2	Fold change
**Acinar cell enzymes**			**Ribosome subunit**		
Alpha amylase 1 (AMY2A)	-0.4	-1.3	Large ribosomal subunit protein uL3m (Mrpl3)	-5.7	-52.0
alpha-amylase 2a5	-0.6	-1.5	Small ribosomal subunit protein uS2m (Mrps2)	-5.6	-48.5
Phospholipase A2 (Pla2g1b)	-0.77	-1.7	Large ribosomal subunit protein uL3-like (Rpl3l)	-5.3	-39.4
Carboxypeptidase A1	-1.1	-2.1	40S ribosomal protein S4 (Rps4l)	-3.3	-9.8
Carboxypeptidase A2	-1.1	-2.1	Large ribosomal subunit protein mL38 (Mrpl38)	-3.3	-9.8
Carboxypeptidase B	-1.1	-2.1	Small ribosomal subunit protein mS34 (Mrps34)	-3.1	-8.6
			Large ribosomal subunit protein mL43 (Mrpl43)	-3.1	-8.6
Phospholipase A2	-0.77	-1.7	Large ribosomal subunit protein uL22m (Mrpl22)	-2.9	-7.5
Trypsin (Prss1)	-2.3	-4.9	Small ribosomal subunit protein uS5m (Mrps5)	-2.9	-7.5
Pancreatic triacylglycerol lipase (Pnlip)	-1.2	-2.3	Small ribosomal subunit protein uS11m (Mrps11)	-2.9	-7.5
Chymotrypsinogen B (Ctrb1)	-1.1	-2.1	Large ribosomal subunit protein uL24m (Mrpl24)	-2.8	-7.0
**Transcription factors**					
GATA4	-5.6	-48.5			
GATA6	-5.1	-34.3			
PDX1	-4.3	-19.7			
**Matrix proteins**			C-C motif chemokine 6 (CCL6)	4.4	21.1
Collagen alpha-4(IV) chain (Col4a4)	1.7	3.2	IL-1ra	3.5	11.3
Collagen, type IV, alpha 6 (Col4a6)	3.8	13.9			
**Adipocytes**					
Perilipin-1 (Plin1)	1.5	2.8			
Perilipin-4 (Plin4)	4.6	24.3			
Long-chain fatty acid transport protein (Slc27a1)	3.4	10.6			
Phospholipid transfer protein (Pltp)	3.0	8.0			
Retinol-binding protein 4 (Rbp4)	1.0	2.0			
